# The catalytic domains of all human KDM5 JmjC demethylases catalyse *N*‐methyl arginine demethylation

**DOI:** 10.1002/1873-3468.14586

**Published:** 2023-02-07

**Authors:** Joanna Bonnici, Razanne Oueini, Eidarus Salah, Catrine Johansson, Christopher J. Schofield, Akane Kawamura

**Affiliations:** ^1^ Chemistry Research Laboratory, Department of Chemistry and the Ineos Oxford Institute for Antimicrobial Research University of Oxford UK; ^2^ Chemistry – School of Natural and Environmental Sciences Newcastle University UK; ^3^ Botnar Research Centre, NIHR Oxford Biomedical Research Unit University of Oxford UK

**Keywords:** 2‐oxoglutarate non‐heme oxygenase, epigenetics, histone *N*‐methyl arginine/lysine demethylase, JmjC‐KDM, post translational modification

## Abstract

The demethylation of *N* ^ε^‐methyllysine residues on histones by Jumonji‐C lysine demethylases (JmjC‐KDMs) has been established. A subset of JmjC‐KDMs has also been reported to have *N* ^ω^‐methylarginine residue demethylase (RDM) activity. Here, we describe biochemical screening studies, showing that the catalytic domains of all human KDM5s (KDM5A‐KDM5D), KDM4E and, to a lesser extent, KDM4A/D, have both KDM and RDM activities with histone peptides. Ras GTPase‐activating protein‐binding protein 1 peptides were shown to be RDM substrates for KDM5C/D. No RDM activity was observed with KDM1A and the other JmjC‐KDMs tested. The results highlight the potential of JmjC‐KDMs to catalyse reactions other than *N* ^ε^‐methyllysine demethylation. Although our study is limited to peptide fragments, the results should help guide biological studies investigating JmjC functions.

## Graphical abstract

Methylation of lysines and arginines in histones is a key epigenetic modification that regulates eukaryotic transcription. A peptide library screen against an extended set of purified human lysine demethylases (KDMs) by mass spectrometry reveals that all KDM5 subfamily members and some KDM4 subfamily members catalyse demethylation of both *N* ^*ε*^‐methyllysines and *N* ^*ω*^‐methylarginines. Not all KDMs tested show arginine demethylase activity.

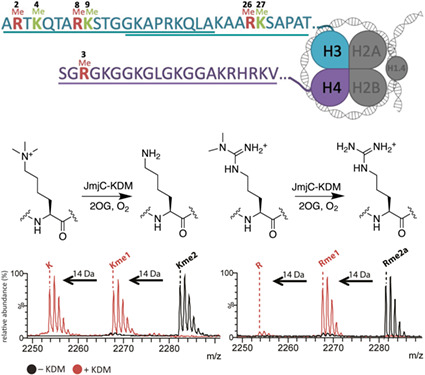

## Abbreviations

**2OG**, 2‐oxoglutarate

**AspH**, aspartyl−/asparaginyl hydroxylase

**FIH**, factor inhibiting hypoxia inducible factor

**FUS**, fused in sarcoma

**G3BP1**, Ras GTPase‐activating protein‐binding protein 1

**hnRNP**, heterogeneous nuclear ribonucleoprotein

**JmjC**, Jumonji‐C

**JmjC‐KDMs**, JmjC‐containing histone demethylases

**KDM**, *N* ^*ε*^‐methyl lysine demethylation

**RDM**, *N* ^*w*^‐methyl arginine demethylation

The *N*‐methylation of lysine and arginine residues in histones is important in the regulation of eukaryotic transcription [1]. Pioneering work completed nearly 50 years ago demonstrated demethylation of histones with concomitant formation of formaldehyde [2], and more recent work has identified specific enzymes that catalyse histone demethylation [3]. Two families of *N* ^*ε*^‐methyl lysine residue demethylases (KDMs) have been identified: the flavin‐dependent lysine specific demethylases (KDM1) and the larger family of 2‐oxoglutarate (2OG) and Fe(II)‐dependent Jumonji C (JmjC) demethylases (KDM2–7). The KDM activities of both these mechanistically distant classes of KDMs are established. The JmjC‐KDMs act on all three *N* ^*ε*^‐methylated states of lysine, whereas the flavin‐dependent demethylases only act on the mono‐ and di‐ *N* ^*ε*^‐methylated states [3, 4].

**Fig. 1:**
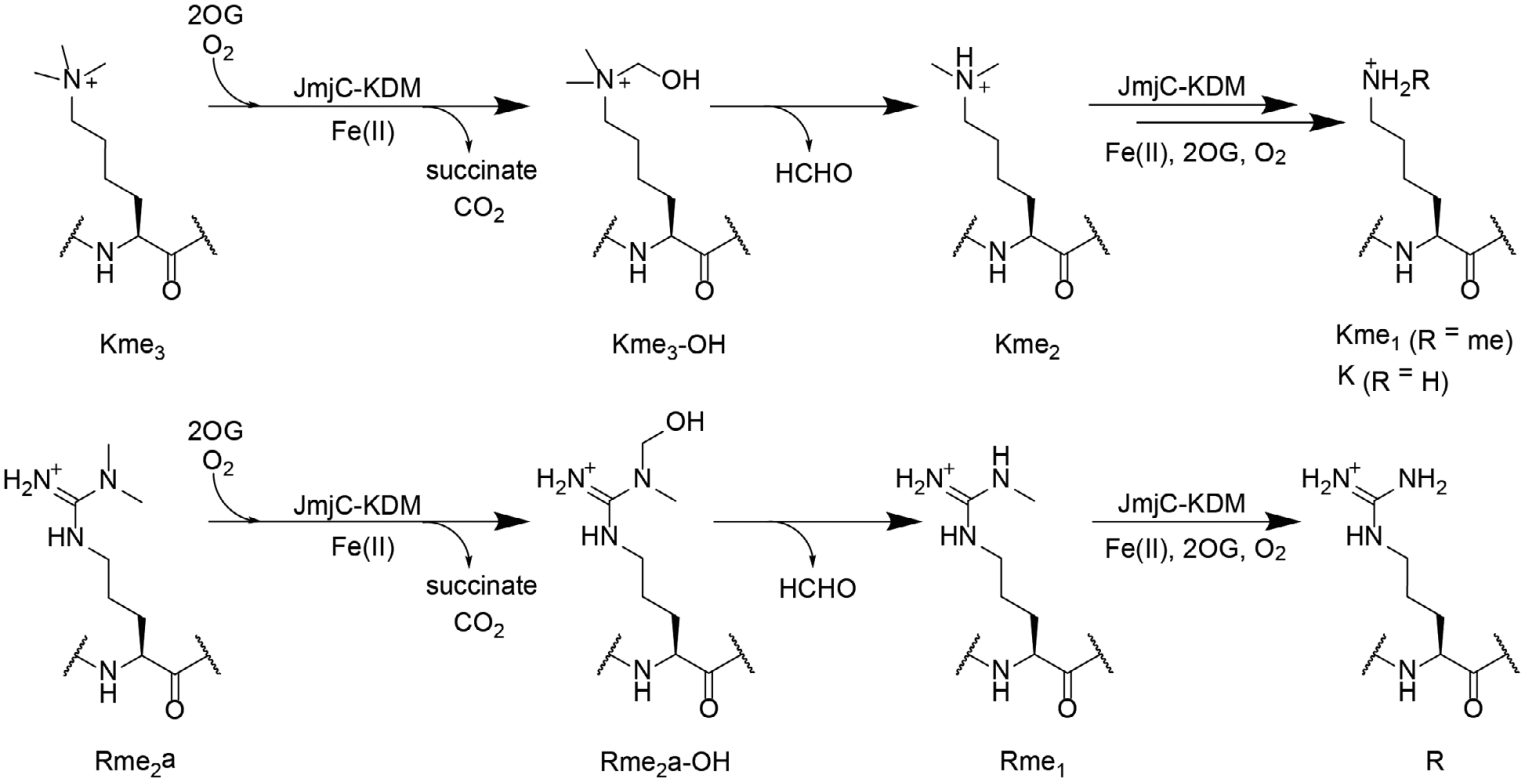
JmjC‐catalysed demethylation of *N*‐methylated lysines and arginines. Demethylation of *N*‐methyllysine (top) and *N*‐methylarginine (bottom) residues involves oxidative decarboxylation of 2OG to form succinate, carbon dioxide, and an unstable hemiaminal, which fragments, releasing formaldehyde and the demethylated product.

The JmjC KDMs belong to the JmjC subfamily of 2OG oxygenases, some of which catalyse hydroxylation of proteins to give stable alcohol products [1]. JmjC‐KDM catalysis also proceeds via hydroxylation, but the available evidence is that the nascent hemiaminal products are unstable, fragmenting to give the demethylated product and formaldehyde (Fig. 1) [5]. 2OG oxygenases catalyse a very wide range of oxidative reactions. In some cases, they have multiple substrates, as is the case for the human JmjC protein hydroxylases JMJD6 and factor inhibiting hypoxia‐inducible factor (FIH) [6, 7, 8, 9]. FIH also catalyses the oxidation of different types of residues, as does the 2OG dependent aspartyl−/asparaginyl‐hydroxylase (AspH), which belongs to a different structural subfamily to the JmjC enzymes [10, 11, 12, 13]. By contrast with the KDM activities of the JmjC‐KDMs, the capacity of these enzymes and other 2OG oxygenases to catalyse demethylation of *N*‐methyl arginine residues (RDM activity) has been less clear.

We have reported initial studies with isolated JmjC‐KDMs demonstrating that some of them have RDM activity [14, 15] and that KDM inhibitors show indiscriminate inhibition of both KDM and RDM activity of the JmjC‐KDMs [15]. Although as yet there is no unequivocal evidence for JmjC enzyme catalysed RDM activity in cells [6, 16, 17], the promiscuity of some 2OG oxygenases, such as FIH, suggests this is a realistic possibility [10, 11, 12, 13]. Here, we describe studies on the RDM activities of the catalytic domains of the KDM4 and KDM5 subfamilies of human JmjC‐KDMs. The results reveal that all the catalytic domains of isolated human KDM5s have both KDM and RDM activities, though (at least with truncated enzymes/ assay conditions used) the RDM activities of the KDM4s are less consistent throughout the subfamily. Combined with earlier work, the results imply that RDM activity is substrate sequence‐ and JmjC‐enzyme‐specific, and in some cases, regions away from the active site may be involved in regulating RDM activity.

## Materials and methods

Unless otherwise stated, reagents and solvents were from Sigma Aldrich (Gillingham, Dorset, UK).

### Enzyme production and purification

Recombinant enzyme constructs with the JmjC catalytic domains were produced and purified via established procedures as summarised in Table S1 and Fig. S1 [18, 19, 20, 21, 22, 23, 24, 25, 26, 27, 28]. Enzymes were analysed by SDS‐PAGE, and concentrations were measured using a Nanodrop ND‐1000 spectrophotometer (Thermo Fisher Scientific, Swindon, Wiltshire, UK). LC–MS validation of protein masses was carried out on selected enzymes using a Xevo G2‐S qTOF machine (Waters, Wilmslow, Cheshire, UK) and a ProSwiftTM RP‐1 S Analytical 4.6 X50 mm SS column (Thermo Fisher Scientific) or with solid phase extraction coupled with quadrupole time‐of‐flight (SPE–qTOF) mass spectrometer (MS). Assays for each KDM were carried out to confirm activities with reported substrates (Fig. S2). KDM4A^1–1064^ was from Active Motif (Waterloo, Belgium) [31457].

### Peptide synthesis and purification

Peptides were synthesised using Liberty Blue (CEM) or Multipep RSi (Intavis Bioanalytical Instruments AG, Koln, Germany) machines depending on the scale/purity required. Fluorenylmethyloxycarbonyl (Fmoc) solid phase chemistry was used with DIC/Oxyma coupling reagents and rink amide 4‐methylbenzhydrylamine (MBHA) polymer‐bound resin. Fmoc‐amino acid monomers were from Merck (Watford, Hertfordshire, UK) or GL Biochem (Shanghai, China). Peptides were prepared as *C*‐terminal amides and stored as lyophilised powders and/or as frozen stock solutions in double distilled (dd) H_2_O at −20 °C. Cleavage from the resin and removal of protecting groups was performed by incubating the resin‐bound protected peptides with 92.5% (v/v) trifluoroacetic acid (TFA), 2.5% (v/v) water, 2.5% (v/v) triisopropylsilane, and 2.5% (v/v) dimethoxybenzene for 3 h. Peptides were then precipitated in ether at 4 °C and centrifuged (4000 rpm, 10 min), and the supernatant was discarded. Masses of the lyophilised peptides produced using the Multipep RSi machine were analysed by MALDI–TOF MS. Peptide synthesis was deemed successful if the peptide was within 1 Da of the calculated mass and used in screening assays without further purification. See Table S2 for a list of peptides used in this study.

Peptides produced using the Liberty Blue machine, for kinetic analyses, were HPLC‐purified (JASCO HPLC) using a GRACE Vydac C18 218TP column (Hichrom, Reading, Berkshire, UK). The gradient of Solvent A [0.1% (v/v) TFA in ddH_2_O] to Solvent B [0.1% (v/v) TFA in acetonitrile] was varied with the peptide to ensure optimal separation. Fractions were analysed by MALDI–TOF MS. Fractions containing purified peptides with the mass of interest were pooled and then lyophilised. Peptide purity was analysed using an LC–MS Vydac 218TP C18 column, Agilent 1200 Series HPLC system coupled to an Agilent 6120 quadrupole mass spectrometer using Solvents A and B as above (using 1 mm peptide), monitored by MS in the positive‐ion mode and by UV (210 nm). mestrenova v12.0.1 (Mestralab Research, Santiao de Compostela, Spain) was used to assess masses and peak areas from the chromatogram. Peptides with purity > 95% were deemed suitable for kinetic analyses. Peptide quantitation was carried out by ^1^H NMR spectroscopy (Bruker AVIII HD 500, Bruker, Coventry, UK), by comparing the integral of a discrete peak from the peptide with that of a 3‐(tri‐methylsilyl)‐propanoic acid (TSP) standard (^1^H chemical shift = 0 ppm). A 58 mm solution of TSP and ~ 50 mm of peptide were dissolved in D_2_O, and combined to give a final concentration of 0.6 mM and ~ 0.5 mM respectively. Spectra were generated using topspin 3.2 (Bruker, Coventry, UK) with manual phase‐correction and baseline correction. Peaks were integrated manually using mestrenova v12.0.1.

### MS‐based enzyme activity assays with peptides

Assays were carried out as reported (see Table S3 for screening conditions used) in either 96‐ or 384‐well plate formats. In brief, an enzyme mixture [containing enzyme and when required, tris(2‐carboxyethyl)phosphine hydrochloride (TCEP) and Triton‐X] and a substrate mixture containing the peptide and cofactors (both at double the required final concentrations) were prepared separately and mixed to initiate reaction. The final total volume for reaction was, in most cases, 10 μL; reactions were quenched using an equal volume of 2% (v/v) formic acid (HCOOH). For negative controls, 5 μL of the enzyme was quenched with 10 μL of 2% (v/v) HCOOH prior to the addition of 5 μL of the substrate mixture. For time course studies, reactions were initiated by mixing equal volumes of the enzyme and substrate mixtures. For each time point, 10 μL of the reaction mixture was withdrawn and quenched with 10 μL of 2% (v/v) HCOOH in water. Unless otherwise stated, time course assays were carried out in triplicate. For screening assays, (unless otherwise stated) 1 μL of peptide (100 μm) was added to 4 μL of a cosubstrate/cofactor mixture prepared at twice the final concentration required and mixed well. Reactions were initiated by addition of 5 μL of the enzyme and incubated for 1 h at room temperature (unless otherwise indicated) and then quenched with 10 μL of 2% (v/v) aqueous HCOOH. The enzyme : peptide ratio was 1 : 5. Positive control assays (with known substrates) were in quadruplicate, while the concurrently assayed potential substrate peptide assays were in duplicate.

For MALDI–TOF MS analyses, each time point was spotted onto either a 96‐ or 384‐spot MALDI target plate and mixed in a 1 : 1 ratio (v/v) with a saturated solution of alpha‐cyano‐4‐hydroxycinnamic acid (CHCA) dissolved in 50% (v/v) acetonitrile, 0.1% 1 : 1 (v/v) aqueous TFA. The dried spots were analysed using either Bruker Microflex™ LRF or a Bruker Autoflex TOF/TOF machines. Data were analysed using FlexAnalysis v3.4 software (Bruker, Coventry, UK); the relative peak intensity (RPI) for the mass corresponding to each methylation state was determined. The RPI was used to calculate the percentage conversion of the substrate to product. Values were normalised using the percentage demethylation values obtained from the 0 min time point or no enzyme control. Small mass variations of ≤ 1 Da were detected due to shifts in calibration; however, when this was observed, it was consistently observed across all peaks within the same spectrum. A mass shift of −14 Da was assigned as corresponding to demethylation.

For LC–MS analyses, samples were analysed from 96‐well assay plates or transferred to LC–MS vials. A Xevo G2‐XS qTOF LC–MS machine controlled using masslynx 4.1 software (Waters) and a Chromolith FastGradient RP‐18 50–2 mm column (Merck) were used. About 10 μL of the sample (5 μm peptide) was injected and a gradient program (Table S4) with a flow rate of 0.3 mL·min^−1^ was run using Solvent A: 0.1% (v/v) HCOOH in ddH_2_O and Solvent B: 0.1% (v/v) HCOOH in acetonitrile. The MS parameters were as follows: positive ionisation, 1.5 kV capillary voltage, 40 V cone voltage, 140 °C source temperature, 50 L·h^−1^ cone gas flow rate, 800 L·h^−1^ desolvation gas flow, and mass range 150–3000 Da. Leucine enkephalin (556.27 Da, 0.05 μg·mL^−1^) was used as a lock‐spray calibrant.

### LC–MS‐based assays with calf thymus histones and KDM4E

Calf thymus histones (0.2 μg·μL^−1^) were incubated (16 h, room temperature) with 9 μm KDM4E (or a no enzyme control), 200 μm 2OG, 100 μm sodium *L*‐ascorbate, 10 μm (NH_4_)_2_Fe(SO_4_)_2_, and 50 mm HEPES (pH 7.5) in final reaction volume of 25 μL. Reactions were quenched with 1 : 1 (v/v) 1% HCOOH for LC–MS analysis, based on previous optimisation [29].

Untreated and KDM4E‐treated calf thymus histone samples were analysed using masslynx 4.1 software and a Xevo G2‐XS qTOF MS machine (Waters) connected to Waters BEH C4 reverse‐phase column (2.1 × 150 mm, 1.7 μm particle size, 300 Å pore size) at 40 °C. Calf thymus histone samples (4 μL, approximately 1000 μg·mL^−1^) were injected with a gradient profile (Table S5) with a flow rate of 0.6 mL·min^−1^ using Solvent A: 0.1% (v/v) HCOOH in ddH_2_O and Solvent B: 0.1% (v/v) HCOOH in acetonitrile. The MS parameters were as follows: positive ionisation mode, 3 kV capillary voltage, 40 V cone voltage, 100 °C source temperature, 50 L·h^−1^ cone gas flow rate, 400 L·h^−1^ desolvation gas flow, and mass range 100–2000 Da. Leucine enkephalin (556.27 Da, 0.05 μg·mL^−1^) was used as a lock‐spray calibrant. Data were deconvoluted using Maxent 1 function using masslynx 4.1 software (Waters).

### FDH‐coupled demethylation assays

Specific activities were measured by monitoring formaldehyde production as reported [22, 30]. Assays were prepared as described above at room temperature, in clear‐bottom black 384‐well plates for 1 h with an enzyme mixture containing 1 μm JmjC‐KDM and 1 μm FDH (see Table S1 for details), and a substrate mixture [peptide, (NH_4_)_2_Fe(SO_4_)_2_, sodium L‐ascorbate, 2OG, and 500 μm β‐nicotinamide adenine dinucleotide (β‐NAD^+^)] in buffer 50 mm HEPES pH 7 containing 0.01% (v/v) Tween (final volume: 50 μL). A PHERAstar FS (BMG Labtech, Aylesbury, Buckinghamshire, UK) plate reader (355 nm excitation and 460 nm emission) was used to measure the NADH fluorescence. A linear regression was fitted to the first‐order region of the reaction and using a formaldehyde calibration curve converted to demethylated product formation (μm). The mean specific activities derived from this data set are given in (substrate) μm·min^−1^·(enzyme) μm ^−1^.

## Results

RDM activity with four JmjC KDMs, that is isolated recombinant KDM3A^515–1317^, KDM4A^1–1064^, KDM5C^1–765^, and KDM6B^1141–1641^ and selected histone peptide substrates, has been reported (Table S6) [14, 15]; the RDM activity was shown to require both 2OG and Fe(II) using KDM6B catalysed demethylation of H3(14–34) R27me2a, a variant (underlined residue) of the natural histone H3K27me3 substrate, as a model reaction [14]. To investigate the broader extent of RDM activity across the human JmjC‐KDM family, we screened a more extensive set of active JmjC‐KDM enzymes than those previously investigated using histone peptides H3(1–15)R2me2a and H4(1–15)R3me2a (see Table S2 for nomenclature). MALDI‐TOF MS‐based assays were used to directly screen for demethylated products (Table S7) [14]. The recombinant JmjC‐KDM panel used in the initial studies comprised representative KDM members from each of the KDM1/3/4/5/6/7 KDM subfamilies, that is KDM3B^882–1761^, KDM4A^1–359^, KDM4B^1–359^, KDM4D^1–358^, KDM5D^1–775^, KDM7A^38–480^ (all 2OG dependent JmjC KDMs), and KDM1A^1–873^, the latter of which is a flavin‐dependent KDM [4] (Tables S3 and S7). To enable comparison with the reported results [14], the previously screened JmjC‐KDMs (KDM3A^515–1317^, KDM4E^1–337^, KDM5C^1–765^, and KDM6B^1141–1641^) were included in the screen, and the assay conditions (Table S3) and peptide lengths were kept consistent (Table S7) [14]. The results of time courses for each of the enzymes with established KDM substrates confirmed efficient substrate turnover after 60 min (Fig. S2). These conditions were then used to screen for RDM activities.

In the case of H3(1–15)R2me2a, evidence for RDM activity was observed with KDM4E^1–337^, KDM5C^1–765^, and KDM5D^1–775^ and, at a lower level, with KDM4D^1–358^. Only KDM4E^1–337^ and KDM5C^1–765^ were observed to catalyse demethylation of H4(1–15)R3me2a (Fig. S3 and S4). No RDM activity was observed for both peptides with KDM4A^1–359^, KDM4B^1–359^, KDM3A^515–1317^, KDM3B^882–1761^, KDM6B^1141–1641^, and KDM7A^38–480^ under conditions where demethylation of an established KDM substrate was complete (Figs S3–S6). No RDM activity was observed for the flavin‐dependent KDM1A^1–873^ (Fig. S6). In all cases where RDM activity was observed, more extensive demethylation of the established *N*‐methylated lysine positive control substrates was observed compared to *N*‐demethylation of the methylated arginine substrates tested, under the same assay conditions (Table S3, Figs S3–S6).

To further investigate the extent of RDM activity across different arginine methylation sites, we screened KDM4A^1–359^, KDM4D^1–358^, KDM4E^1–337^, and KDM5C^1–765^ against an extended set of histone H3 and histone H4 peptides with biologically relevant methylated arginine sites (Table S8) [31]. H3(1–21)K9me3 and H3(1–21)K4me3 were included as positive controls for the KDM4 and KDM5 enzymes, respectively. As observed in the previous screen, in all cases, more demethylation of the methylated lysine substrates was observed compared to the methylated arginine substrates with 60‐min incubations (Table S8).

In the case of KDM5C, in addition to the RDM activity that was previously observed, i.e. demethylation of H3(1–15)R8me2a, H3(1–15)R8me2s, H4(1–15)R3me2a, and H4(1–15)R3me2s (Table S6) [14], RDM activity was additionally observed with H3(1–20)R8me1 and H4(1–20)R3me1 (Figs S7 and S8). For KDM5C (as for KDM4D), Rme2a appears to be preferred for RDM activity compared to Rme2s; more extensive demethylation was observed when KDM5C was incubated with H3(1–20)R8me2a and H4(1–20)R3me2a compared to H3(1–20)R8me2s and H4(1–20)R3me2s, respectively (Figs S7–S9).

**Fig. 2:**
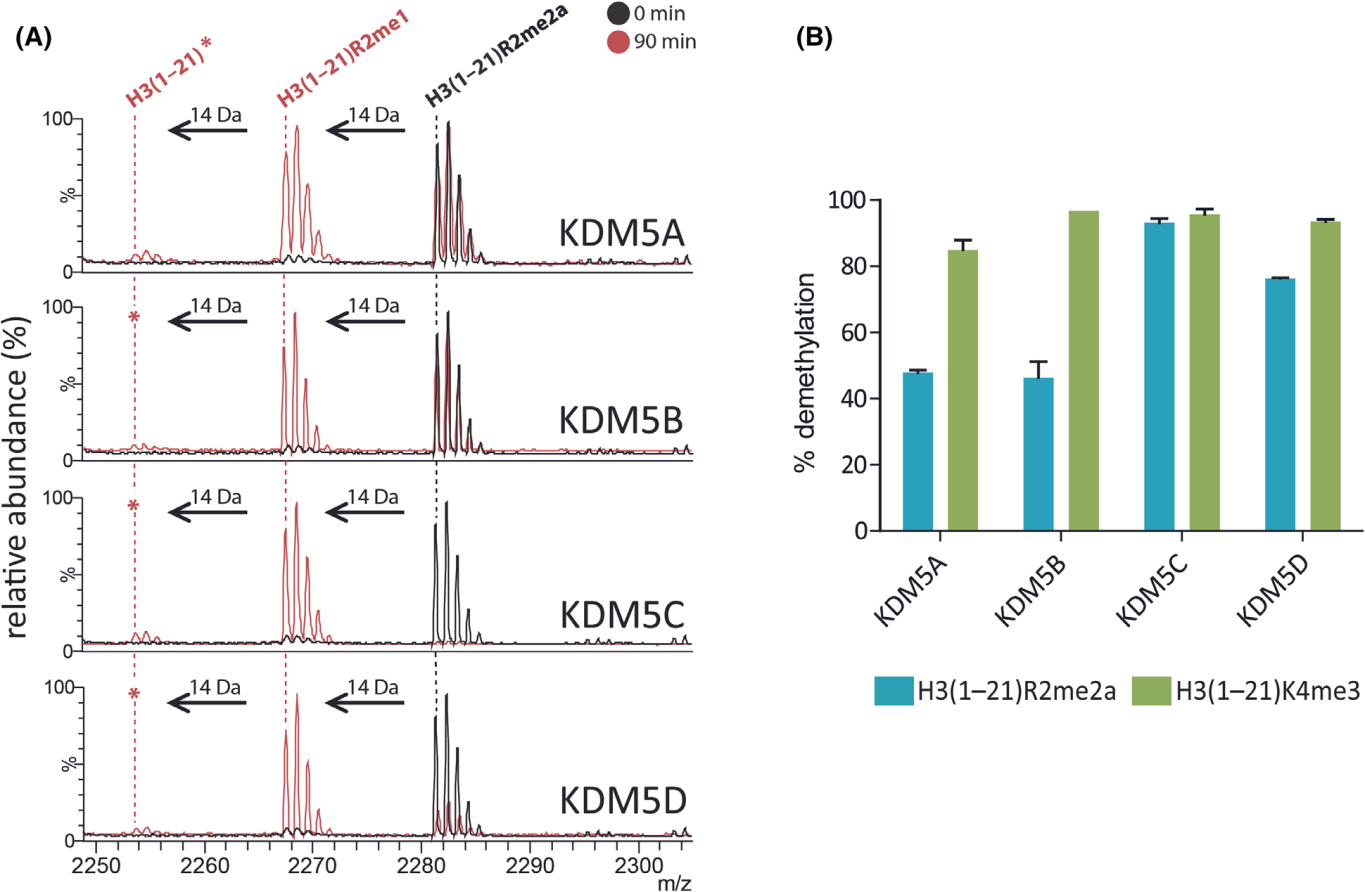
All KDM5 subfamily members demethylate *N*‐methyl‐arginines. (A) MALDI–TOF MS analyses reveal −14 Da peaks from H3(1–21)R2me2a corresponding to removal of methyl groups following incubation with KDM5A‐D (90 min, red) at 37 °C. Charge state of labelled ions: [MH]^+^. *: low‐level demethylation (< 10% relative abundance). (B) Comparison of KDM5A‐D‐catalysed demethylation of H3(1–21)R2me2a and H3(1–21)K4me3 under the same assay conditions (37 °C, 90 min). Average demethylation (%) ± SD (*n* = 3 independent assays) are shown for each substrate/enzyme combination. See Table S3 for assay conditions.

Next, we explored the RDM activities across all of the KDM5 and KDM4 subfamily members. Since the results revealed that both KDM5C and KDM5D clearly exhibited RDM activity, we investigated whether this is the case for other KDM5 family members. Indeed, all the tested KDM5 members showed RDM activity with H3(1–21)R2me2a (Fig. 2A). After 90‐min incubation, KDM5A and KDM5B catalysed ~ 50% demethylation of H3(1–21)R2me2a, KDM5C catalysed ~ 90% demethylation, and KDM5D catalysed ~ 80% demethylation, under the same conditions (Fig. 2A,B). In all cases, except for KDM5C (where complete demethylation of both substrates was observed), with 90‐min incubations, more of the positive control H3(1–21)K4me3 peptide was turned over compared to H3(1–21)R2me2a (Fig. 2B, Fig. S10).

A different trend was, however, observed with the KDM4 subfamily. With the peptide library screens, the catalytic domain of KDM4A (KDM4A^1–359^) did not manifest detectable RDM activity (Tables S7 and S8). The same result was obtained using a higher concentration of KDM4A and H3(1–20)R2me2a or H3(1–15)R9me2a, a variant (underlined residue) of the natural histone H3K9me3 substrate of KDM4A [32], with an *N*‐methylated arginine at K9 (Fig. S11). This observation contrasts with RDM activity observed with H3(1–15)R2me2a using full‐length KDM4A^1–1064^, which was purified from transfected mammalian cells [14]. To investigate this difference, KDM4A^1–1064^ and KDM4A^1–359^ (produced via baculovirus and *Escherichia coli* expression systems, respectively) were incubated with H3(1–21)R2me2a and H3(1–21)R9me2a; only low levels of RDM activity, if any, were observed with both constructs (Fig. S12).

**Fig. 3:**
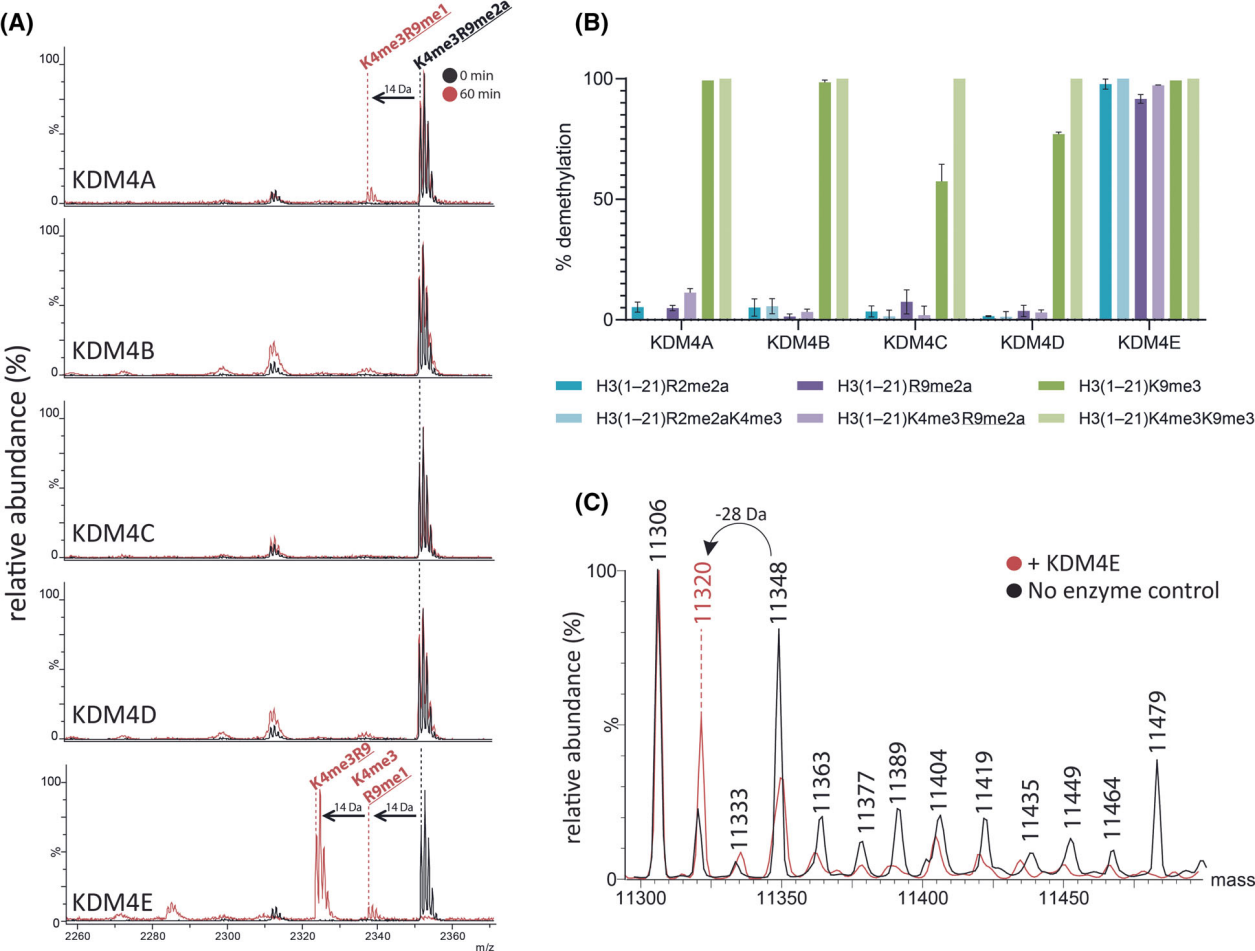
Some KDM4 subfamily members demethylate *N*‐methyl‐arginines. (A) MALDI–TOF MS analyses reveal −14 Da peaks corresponding to the removal of methyl groups of H3(1–21)K4me3R9me2a following incubation (60 min) with KDM4A and KDM4E. Charge state of labelled ions: [MH]+. (B) Comparison of KDM4A‐E catalysed demethylation of H3(1–21)R2me2a (cyan), H3(1–21)R2me2aK4me3 (purple), H3(1–21)R9me2a (red), H3(1–21)K4me3R9me2a (blue), H3(1–21)K9me3 (green), and H3(1–21)K4me3K9me3 (orange) under the same assay conditions (37 °C, 60 min). Average demethylation (%) ± SD (*n* = 3 independent assays) are shown for each substrate/enzyme combination. See Table S3 for assay conditions. (C) Representative deconvoluted LC–MS for 16‐h incubation of KDM4E with calf histone H4 showing a −28 Da mass shift consistent with removal of two methyl groups. *n* = 3 (independent assays).

Next, we explored the effect of ‘combinatorial’ PTMs on RDM activity, because it has been reported that the presence of H3K4me3 increases H3K9me3 demethylation cataslyed by the KDM4s (1.5–2 fold increase in *k*_cat_/*K*_M_ for catalytic KDM4A/C construct, up to 17‐fold for full length KDM4A) [33]. The KDM/RDM activities on H3K9me3, H3R2me2a, and H3R9me2a in the presence and absence of H3K4me3 mark were tested across all KDM4 subfamily members (catalytic domains); note that previous work has indicated that H3K4me3 is not demethylated by the KDM4 demethylases [34, 35, 36], consistent with our own work [37]. Consistent with the previous findings [33], the presence of H3K4me3 enhanced demethylation activity of H3(1–21)K9me3, in particular, where ~ 43% and ~ 23% increases in KDM activities were observed for KDM4C and KDM4D, respectively (Fig. 3A,B, Fig. S13A). In the case of H3R2me2a, the presence of H3K4me3 did not improve RDM activity of KDM4s for H3(1–21)R2me2a, with little (< 10%) or no demethylation observed across the KDM4 subfamily, with the exception of KDM4E where > 95% demethylation was observed for both peptides (Fig. 3B, Fig. S13B). However, the presence of the H3K4me3 mark moderately increased the RDM activity of KDM4A with H3R9me2a (from 5% to 11% demethylation) (Fig. 3B, Fig. S13C). Similar RDM activity was observed for the full‐length KDM4A construct (17% demethylation at 60 min) (Fig. S12). This trend was not observed with KDM4B, KDM4C, and KDM4D. Thus, although care should be taken in interpreting low levels of activity, at least under our assay conditions, the presence of the K4me3 modification would appear to affect KDM and RDM activities at K9 to different extents for different KDM4 subfamily members.

With the peptide library screen, KDM4D manifested low levels of RDM activity with H3(1–20)R2me2a and H3(1–20)R2me2s, that is at a similar level to that observed with H3(1–15)R2me2a (Figs S3, S8 and S9). Prolonged incubation (120 min) with an increased amount (from 2 to 10 μm) of KDM4D with H3(1–21)R2me2a did not increase the low levels (~ 7%) of RDM activity. The level of RDM activity with KDM4D was much lower than that of KDM4E (~ 83% demethylation under the same conditions) (Fig. S14).

Evidence that the RDM activity of the KDM4 enzymes on peptides is relevant with intact histones came from incubation of KDM4E with histone H4 extracted from calf thymus, which is known to be modified by two methylations at H4R3 [31]. We observed a −28 Da mass shift, consistent with two demethylations. Whilst this could be the result of demethylation elsewhere, because no other histone H4 modifications have been identified to be potential KDM4 substrates other than H4R3 [14], it seems likely that this mass shift reflects RDM activity by KDM4E at H4R3 (Fig. 3C).

To investigate whether the levels, if any, of RDM activities correlate with the KDM activity for different KDM4 subfamily members and to test whether the enzyme activities were comparable to those previously reported [37], we carried out assays comparing the specific activities of all KDM4 subfamily members (A‐E) with the reported H3K9me3 KDM4 substrates of different lengths, that is, H3(1–15)K9me3 and H3(1–21)K9me3, using a reported formaldehyde dehydrogenase coupled (FDH) assay [22, 30]. This technique was chosen to compare results with previous reports and because it consumes less reagents compared to the MS‐based assays.

KDM4A had the highest observed specific activity [7.16 and 9.01 μm·min^−1^·μm ^−1^, for H3(1–15)K9me3 and H3(1–21)K9me3, respectively]; KDM4D and KDM4E had very similar KDM activities (KDM4D: 5.66 and 7.06 μm·min^−1^·μm ^−1^, respectively; KDM4E: 7.24 and 6.69 μm·min^−1^·μm ^−1^ respectively), followed by KDM4B (2.67 and 3.09 μm·min^−1^·μm ^−1^), and KDM4C had the lowest observed activity (1.46 and 2.01 μm·min^−1^·μm ^−1^). These results are comparable with previous reports comparing KDM4 activity with H3K9me3 peptides where KDM4A, KDM4D, and KDM4E have reportedly similar levels of activity, followed by KDM4B and KDM4C with the lowest level of catalytic activity [37]. Given that previous specific activities reported using the same construct and assay conditions for KDM4E showed H3(1‐15)R2me2a demethylation to be > 40‐fold slower than H3(1‐15)K9me2 [15], it is possible that the RDM activity is significantly lower (if present at all) compared to KDM activity across the KDM4 family. However, the available evidence is that KDM4E most efficiently catalyses arginine demethylation amongst the KDM4s, despite having similar KDM activity compared to other KDM4 subfamily members.

Sequence alignment of both the JmjN and JmjC domains of all five KDM4 enzymes shows high levels of similarity (Fig. S15). Despite this and the likelihood that the RDM and KDM reactions proceed via similar mechanisms (Fig. 1), the results imply that the variations in RDM activity for isolated KDM4 family members reflect intrinsic differences in RDM substrate activity/selectivity, that are not reflected in the differences in the KDM activities as observed with established *N*‐methylated lysine substrates (Fig. S16).

**Fig. 4:**
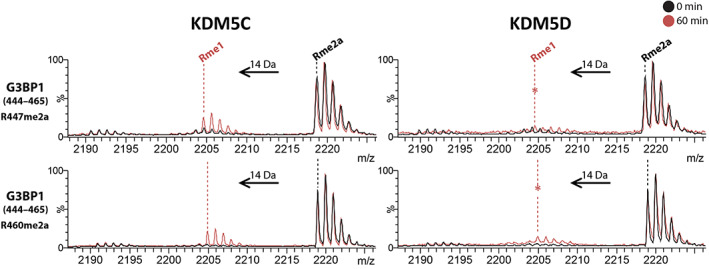
KDM5 demethylate *N*‐methyl‐arginines of non‐histone proteins. Representative MALDI–TOF MS following incubation of KDM5C (left) and KDM5D (right) with G3BP1(444–465)R447me2a (top), and G3BP1(444–465)R460me2a (bottom) showing −14 Da decreases in mass corresponding to partial demethylation (*n* = 2, technical duplicates). (*) low‐level demethylation (< 10% relative abundance). *Y*‐axis: relative abundance (%). Refer to Table S3 for assay conditions.

*N* ‐Methylations of lysine‐ and arginine‐ residues are reported in multiple non‐histone human proteins [38, 39, 40, 41, 42, 43, 44, 45, 46, 47, 48, 49], and there is some evidence KDMs may act on non‐histone proteins [50, 51, 52, 53, 54, 55, 56]. There is evidence that JmjC‐KDMs, at least in isolated form, can also manifest RDM activity on non‐histone peptide fragments: KDM4E is reported to catalyse demethylation of heterogeneous nuclear ribonucleoprotein (hnRNP) at R296me2a, and KDM5C is reported to catalyse demethylation of TP53‐binding protein 1 (53BP1) at R1401me2a [14]. To explore potential non‐histone JmjC RDM substrates, we incubated our screening panel of demethylases (KDM1A, KDM3A/B, KDM4A/D/E, KDM5C/D, KDM6B, and KDM7A) (Table S7) with peptides from proteins reported to have methylated arginines, that is Ras GTPase‐activating protein‐binding protein 1 (G3BP1, a component of cytoplasmic stress granules (SGs) which is methylated at R435, R447, and R460 by PRMT1, PRMT5, and PRMT8) [48] and fused in sarcoma (FUS, which is methylated at R498 and R503) [47]. Incubations with G3BP1(430–443)R435me2a, G3BP1(431–451)R447me2a, G3BP1(444–465)R447me2a, and G3BP1(444–465)R460me2a led to the observation that KDM5C and KDM5D catalyse (to different extents) demethylation of the Ras G3BP1 peptides, at R447 and R460 (Fig. 4, Figs S17 and S18). FUS(488‐513)R498me2aR503me2a, FUS(488‐513)R498me2a, and FUS(488‐513)R503me2a peptides were not observed to be RDM substrates under the conditions tested (Fig. S19). The RDM activities with non‐histone substrates thus appear to be sequence‐specific, as evidenced by the sequence alignment of the potential substrates tested and shown in Table S9.

## Discussion

**Fig. 5:**
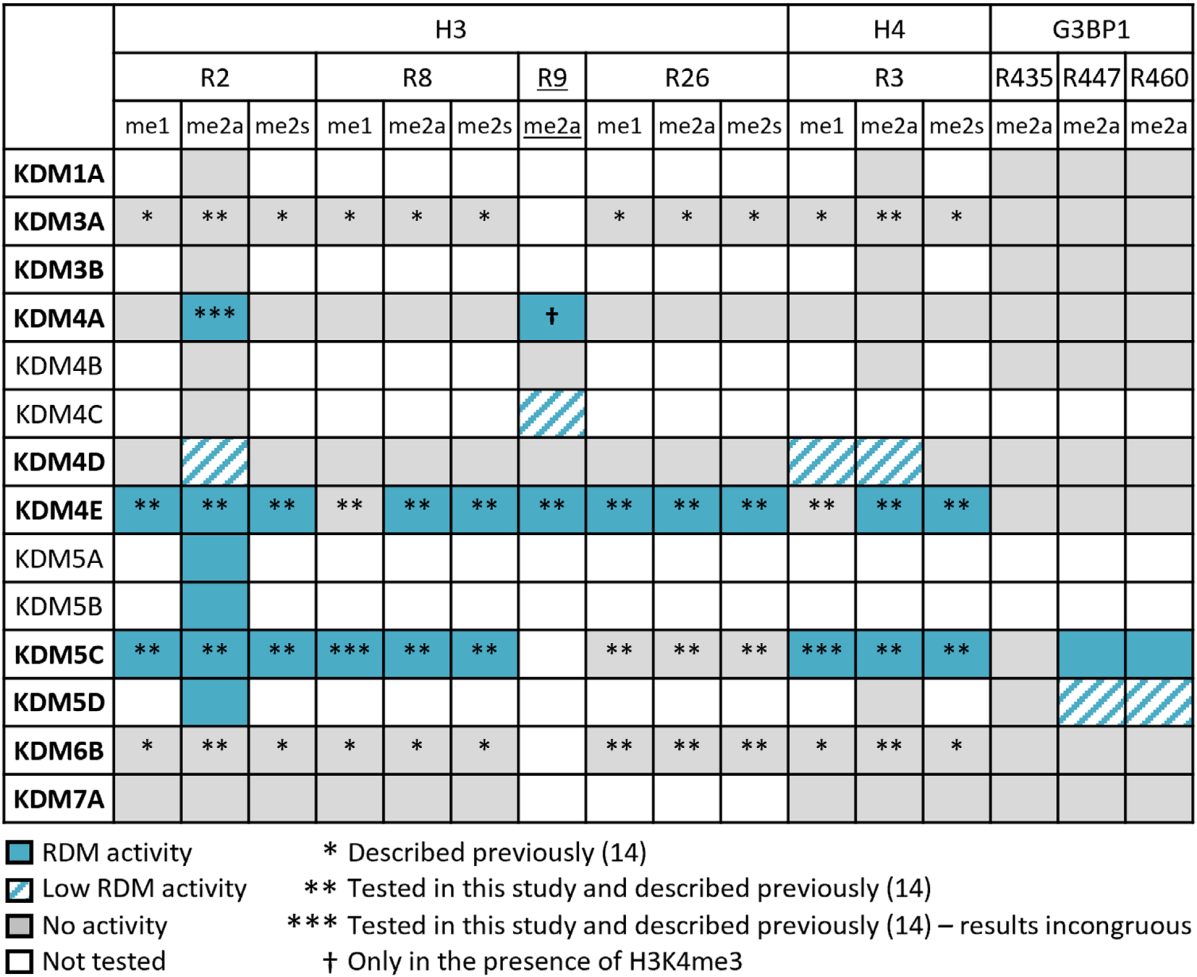
Summary of RDM activities with histone H3 and H4 peptides and non‐histone peptides. RDM activity of H3R2me2a has been observed previously with KDM4A(1–1064) [14], but was not observed with differently prepared KDM4A(1–1064) or KDM4A(1–359) used in this work. KDM5C RDM activity with H3R8me1 and H4R3me1 was observed here with a 20‐mer peptide, but not in a previous study with a 15‐mer peptide [14]. Additional observations from this work: RDM activity with KDM4A and H3R9me2a (in the presence of H3K4me3), KDM4C and H3R9me2a, KDM4D and H3R2me2a, H4R3me2a, H4R3me1; KDM5A and KDM5B and H3R2me2a; KDM5C and G3BP1R447me2a and G3BP1R460me2a; and KDM5D and H3R2me2a, G3BP1R447me2a and G3BP1R460me2a. RDM activity observed either in this study or previously or in both is in blue. Low‐level RDM activity is indicated with dashed blue (< 10% product formation). All methylated arginine histone substrates have been reported [31] except for H3R9me2a which is a variant (underlined) of the natural histone H3K9me3 substrate of the KDM4s. Note that not all putative methylated arginine histone substrates tested are shown – only those yielding positive results. Enzymes in bold were part of initial screens, and the rest were used in follow‐up investigations.

The results presented here clarify the potential of JmjC‐KDMs to have RDM activity, as shown by studies both on histone fragment peptides, on histone H4 from calf thymus (with KDM4E), and non‐histone substrates (G3BP1 with KDM5C and KDM5D; summarised in Fig. 5). Notably, our results reveal that the catalytic domains of *all* identified human KDM5 members (A–D) have dual KDM and RDM activities in their isolated forms. In the case of KDM5C, demethylation was observed with the 20‐residue peptides H3(1–20)R8me1 (Fig. S7) and H4(1–20)R3me1 (Fig. S8); however, RDM activity was not observed in our previous report using 15‐mer peptide fragments [14]. This observation is in accord with studies showing that different substrate lengths can affect 2OG oxygenase‐dependent protein hydroxylase activity [57, 58, 59].

The results with the other JmjC‐KDM subfamily members were less clear. Though we did not see any evidence for RDM activity with the flavin‐dependent KDM1A, within the KDM4 JmjC subfamily, we observed clear RDM activity with KDM4E, though to a substantially lesser extent with the other members of the KDM4 subfamily. We did not observe RDM activity with representatives of other tested JmjC‐KDM subfamilies (KDM3A/B, KDM6B, and KDM7A), though RDM (and indeed other) activities cannot be ruled out with protein substrates.

KDM4A is a particularly interesting case. We observed RDM activity with histone fragment H3(1–15)R2me2a when full length, KDM4A^1–1064^ was prepared from human cells [14]. However, substantially less RDM activity was observed with full‐length KDM4A^1–1064^ prepared using a baculovirus expression system and with a truncated construct of KDM4A prepared in *E. coli* (KDM4A^1–359^). Clear evidence for RDM activity with H3(1–21)R9me2a and KDM4A^1–1064^ and KDM4A^1–359^ was only observed when K4me3 was present, a modification that is not a substrate for the KDM4 enzymes [34, 35, 36], but which is reported to enhance the catalytic efficiency of KDM4A and KDM4C at K9me3 [33]. Whilst enzyme–substrate interactions outside of the catalytic domain may contribute towards KDM and RDM activities (e.g. PHD‐finger or Tudor reader domains in KDM4A), we have also shown clear demethylation of Rme2a within the sequence context of high‐affinity *de novo* cyclic peptide CP2(R6me2a) using the truncated KDM4A^1–359^ construct [60]. The combined results thus suggest that, at least in some cases, factors away from the immediate active site region, both within and outside of the catalytic domain, can influence (to different extents) the JmjC KDM and RDM activities, at least of KDM4A. Such factors could be complex as modelling studies based on crystal structures have revealed the potential of correlated motions in KDM, including KDM4A catalysis [61]. Such notions have been shown experimentally for the oxidase isopenicillin *N* synthase, which is structurally and mechanistically related to the 2OG oxygenases [62].

Modelling studies with KDM4A/E [63] and crystallographic studies with KDM4A [14] imply that the chemical mechanisms of KDM and RDM catalysis are similar, that is, both proceed via initial *N*‐methyl group hydroxylation. However, the experimental results presented here and previously [14] also demonstrate that, at least in some cases, sequence selectivity can differ for KDM and RDM activities for the same JmjC‐KDM enzyme, as exemplified by comparative studies on KDM4 enzymes, where the presence of H3K4me3 had different effects on different KDM and RDM reactions (Fig. 3B, Figs S12 and S13). The effect of the presence of H3K4me3 on demethylation activity was most clear in the case of KDM activities of KDM4C and KDM4D with H3(1–21)K9me3 and H3(1–21)K4me3K9me3 where clear evidence for increased KDM activities was accrued (Fig. 3A,B, Fig. S13). However, in the cases of the other KDM4 substrate combinations tested, no clear evidence for substantially increased (or decreased) activities was observed (except for KDM4A and H3(1–21)R9me2a and H3(1–21)K4me3R9me2a).

Together with previous studies, our results expand the observation that some, but not all, JmjC‐KDMs can catalyse RDM reactions. As is the case for lysine demethylation, the efficiencies of the *N*‐methyl‐arginine demethylation reactions (with histone or non‐histone substrates) are sequence‐ and enzyme‐dependent. The changes in expression levels of KDM4s and KDM5s and their activities have been linked to cancer development and progression, as well as cancer drug resistance, and tolerance. We hope our studies with isolated JmjC KDMs will guide studies on the roles of JmjC oxygenases in healthy physiology and disease, including potential RDM activities as well as enabling development of JmjC‐oxygenase inhibitors [15] – both KDM4s and KDM5s are being pursued as therapeutic targets (see, e.g. [64, 65, 66]). It is important to state that although plausible, acquiring unequivocal evidence for JmjC catalysed RDM reactions in cells is challenging. In part, this is because of the essential requirement for prior arginine‐methylation which complicates quantitative analyses, and in part because of limitations in current antibody and MS‐based assays. Further, care should be taken in assuming that the selectivities reported here will apply in a cellular context, where JmjC catalysis occurs on chromatin and likely occurs within the context of multicomponent complexes. Nonetheless, our *in vitro* results showing that all identified KDM5 family members have RDM activity on histone peptide sequences suggests that future studies on the *in vivo* roles of KDM5 *N*‐methyl arginine residue demethylation are of interest.

## Supporting information


**Fig. S1.** Characterisation of purified enzymes.
**Fig. S2.** Characterisation of enzyme activities with established substrates.
**Fig. S3.** Initial screening of KDM4A/B/D/E with H3(1–15)R2me2a and H4(1–15)R3me2a.
**Fig. S4.** nitial screening of KDM5C/D with H3(1–15)R2me2a and H4(1–15)R3me2a.
**Fig. S5.** Initial screening of KDM3A/B with H3(1–15)R2me2a and H4(1–15)R3me2a.
**Fig. S6.** Initial screening of KDM1A, KDM6B and KDM7A with H3(1–15)R2me2a and H4(1–15)R3me2a.
**Fig. S7.** Representative MALDI–TOF MS following 60‐minute incubation of KDM5C with H3R8 peptides (from top to bottom) H3(1–20)R8me2a, H3(1–20)R8me2s, and H3(1–20)R8me1 peptides from the AltaBioscience peptide library (Set 5).
**Fig. S8.** Representative MALDI–TOF MS following 60‐minute incubation of (left) KDM4D and (right) KDM5C with H4R3 peptides (from top to bottom): H4(1–20)R3me2a, H4(1–20)R3me2s, and H4(1–20)R3me1 peptides from the AltaBioscience phosphorylation and arginine methylation histone library (Set 5).
**Fig. S9.** Representative MALDI–TOF MS following 60‐minute incubation of KDM4D with H3R2 peptides (from top to bottom): H3(1–20)R2me2a, H3(1–20)R2me2s, and H3(1–20)R2me1 peptides from AltaBioscience peptide library (Set 5).
**Fig. S10.** Positive control for experiment in Figure 2A (main text).
**Fig. S11.** RDM activity was not observed with a higher concentration of KDM4A^1–359^.
**Fig. S12.** KDM4A^1–1064^ and KDM4A^1–359^ have similar RDM activities.
**Fig. S13.** (A) KDM4s and H3(1–21)K9me3 and H3(1–21)K4me3K9me3. (B) KDM4s and H3(1–21)R2me2a and H3(1–21)R2me2aK4me3. (C) KDM4s and H3(1–21)R9me2a and H3(1–21)K4me3R9me2a.
**Fig. S14.** Comparison of RDM activities of KDM4E and KDM4D with H3(1–21)R2me2a.
**Fig. S15.** Analysis of the KDM4 sequences using Clustal omega.
**Fig. S16.** Comparison of demethylation of H3(1–15)K9me3 (green) and H3(1–21)K9me3 (blue) as catalysed by 1 μM of KDM4A, KDM4B, KDM4C, KDM4D, and KDM4E.
**Fig. S17.** G3BP1 peptides with KDM5C and KDM5D.
**Fig. S18.** G3BP1 peptides with KDM4E.
**Fig. S19.** FUS peptides with KDM4A, KDM4B, KDM4E, KDM5C and KDM5D.
**Table S1.** Summary of constructs and expression systems used.
**Table S2.** Peptides used.
**Table S3.** Summary of MALDI–TOF MS assay conditions for enzymes optimised for substrate screening.
**Table S4.** Conditions for LC–MS‐based activity assays using Chromolith FastGradient RP‐18 50‐2 mm column (Merc) and a Xevo G2‐XS Quadrupole Time‐of‐Flight (qTOF) MS machine.
**Table S5.** Conditions for calf thymus histone LC–MS‐based activity assays using Waters BEH C4 reversed phase column (2.1 × 150 mm, 1.7 μm particle size, 300 A pore size) with A Xevo G2‐XS qTOF MS machine.
**Table S6.** Summary of results for incubations of histone H3 and H4 fragments with Nmethylated arginines previously reported with KDM3A^515–1317^, KDM4A^1–1,064^, KDM4E^1–337^, KDM5C^1–765^, and KDM6B^1141–1,641^.
**Table S7.** Summary of screen of histone and non‐histone peptides with a panel of KDMs for RDM activity.
**Table S8.** Summary of results with unmodified, methylated, and citrullinated histone H3 and H4 peptides from the histone peptide library (AltaBiosciences Set 5) screened with KDM4A, KDM4D, KDM4E, and KDM5C.
**Table S9.** Comparison of sequences surrounding potential arginine substrates of the KDMs.

## Data Availability

The data that support the findings of this study are available in the Supporting Information of this article.
