# Supplementary material for: The catalytic domains of all human KDM5 JmjC demethylases catalyse N‐methyl arginine demethylation
Source: FEBS Lett. 2023 Feb 7;597(7):933–46. doi: 10.1002/1873-3468.14586 (PMC10952680; doi:10.1002/1873-3468.14586)
Supplement: Supplementary file 1 — Fig. S1. Characterisation of purified enzymes. Fig. S2. Characterisation of enzyme activities with established substrates. Fig. S3. Initial screening of KDM4A/B/D/E with H3(1–15)R2me2a and H4(1–15)R3me2a. Fig. S4. nitial screening of KDM5C/D with H3(1–15)R2me2a and H4(1–15)R3me2a. Fig. S5. Initial screening of KDM3A/B with H3(1–15)R2me2a and H4(1–15)R3me2a. Fig. S6. Initial screening of KDM1A, KDM6B and KDM7A with H3(1–15)R2me2a and H4(1–15)R3me2a. Fig. S7. Representative MALDI–TOF MS following 60‐minute incubation of KDM5C with H3R8 peptides (from top to bottom) H3(1–20)R8me2a, H3(1–20)R8me2s, and H3(1–20)R8me1 peptides from the AltaBioscience peptide library (Set 5). Fig. S8. Representative MALDI–TOF MS following 60‐minute incubation of (left) KDM4D and (right) KDM5C with H4R3 peptides (from top to bottom): H4(1–20)R3me2a, H4(1–20)R3me2s, and H4(1–20)R3me1 peptides from the AltaBioscience phosphorylation and arginine methylation histone library (Set 5). Fig. S9. Representative MALDI–TOF MS following 60‐minute incubation of KDM4D with H3R2 peptides (from top to bottom): H3(1–20)R2me2a, H3(1–20)R2me2s, and H3(1–20)R2me1 peptides from AltaBioscience peptide library (Set 5). Fig. S10. Positive control for experiment in Figure 2A (main text). Fig. S11. RDM activity was not observed with a higher concentration of KDM4A1–359. Fig. S12. KDM4A1–1064 and KDM4A1–359 have similar RDM activities. Fig. S13. (A) KDM4s and H3(1–21)K9me3 and H3(1–21)K4me3K9me3. (B) KDM4s and H3(1–21)R2me2a and H3(1–21)R2me2aK4me3. (C) KDM4s and H3(1–21)R9me2a and H3(1–21)K4me3R9me2a. Fig. S14. Comparison of RDM activities of KDM4E and KDM4D with H3(1–21)R2me2a. Fig. S15. Analysis of the KDM4 sequences using Clustal omega. Fig. S16. Comparison of demethylation of H3(1–15)K9me3 (green) and H3(1–21)K9me3 (blue) as catalysed by 1 μM of KDM4A, KDM4B, KDM4C, KDM4D, and KDM4E. Fig. S17. G3BP1 peptides with KDM5C and KDM5D. Fig. S18. G3BP1 peptides with KDM4E. Fig. S19. FUS peptides with KDM4A, KDM4B, KDM [file FEB2-597-933-s001.pdf]

Supplementary Information

The catalytic domains of all human KDM5 JmjC demethylases catalyse *N*-methyl arginine demethylation

Joanna Bonnici[1,2], Razanne Oueini[1], Eidarus Salah[1], Catrine Johansson[3], Christopher J Schofield[1*], and Akane Kawamura[1,2*]

[1] Chemistry Research Laboratory, Department of Chemistry and the Ineos Oxford Institute for Antimicrobial Research, University of Oxford, Oxford OX1 3TA, United Kingdom

[2] Chemistry - School of Natural and Environmental Sciences, Newcastle University, Newcastle Upon Tyne, NE1 7RU, United Kingdom

[3] Botnar Research Centre, NIHR Oxford Biomedical Research Unit, University of Oxford, Oxford, OX3 7LD, United Kingdom

*Correspondence:
christopher.schofield@chem.ox.ac.uk; akane.kawamura@newcastle.ac.uk

Running title: *N*-methyl arginine demethylation catalysed by JmjC-KDMs

Key words: Epigenetics, JmjC 2-oxoglutarate non-heme oxygenase, histone N-methyl arginine / lysine demethylase, regulation of transcription, post translational modification

Table of Contents

Figure S1 Characterisation of purified enzymes. 3

Figure S2 Characterisation of enzyme activities with established substrates. 4

Figure S3 Initial screening of KDM4A/B/D/E with H3(1–15)R2me2a and H4(1–15)R3me2a. 6

Figure S4 Initial screening of KDM5C/D with H3(1–15)R2me2a and H4(1–15)R3me2a. 7

Figure S5 Initial screening of KDM3A/B with H3(1–15)R2me2a and H4(1–15)R3me2a. 8

Figure S6 Initial screening of KDM1A, KDM6B and KDM7A with H3(1–15)R2me2a and H4(1–15)R3me2a. 9

Figure S7 Representative MALDI–TOF MS following 60-minute incubation of KDM5C with H3R8 peptides 10

Figure S8 Representative MALDI–TOF MS following 60-minute incubation of (left) KDM4D and (right) KDM5C with H4R3 peptides 11

Figure S9 Representative MALDI–TOF MS following 60-minute incubation of KDM4D with H3R2 peptides 12

Figure S10 Positive control for experiment in Figure 2A (main text). 13

Figure S11 No RDM observed at a higher concentration of KDM4A$^{1–359}$. 14

Figure S12 No difference in RDM activity of KDM4A$^{1–1064}$ and KDM4A$^{1–359}$ 15

Figure S13A KDM4s and H3(1–21)K9me3 and H3(1–21)K4me3K9me3. 16

Figure S13B KDM4s and H3(1–21)R2me2a and H3(1–21)R2me2aK4me3. 17

Figure S13C KDM4s and H3(1–21)R9me2a and H3(1–21)K4me3R9me2a. 18

Figure S14 Comparison of RDM activities of KDM4E and KDM4D with H3(1–21)R2me2a. 19

Figure S15 Analysis of the KDM4 sequences using Clustal omega. 20

Figure S16 Comparison of demethylation of H3(1–15)K9me3 (green) and H3(1–21)K9me3 (blue) as catalysed by 1 µM of KDM4A, KDM4B, KDM4C, KDM4D, and KDM4E 21

Figure S17 G3BP1 peptides with KDM5C and KDM5D 22

Figure S18 G3BP1 peptides with KDM4E. 23

Figure S19 FUS peptides KDM4A, KDM4B, KDM4E, KDM5C and KDM5D 24

Table S1 Summary of constructs and expression systems used. 25

Table S2 Peptides used. 27

Table S3 Summary of MALDI–TOF MS assay conditions for enzymes optimised for substrate screening. 28

Table S4 Conditions for LC–MS-based activity assays 29

Table S5 Conditions for calf thymus histone LC–MS-based activity assays 30

Table S6 Summary of results for incubations of histone H3 and H4 fragments with N-methylated arginines previously reported with KDM3A$^{515–1317}$, KDM4A$^{1–1,064}$, KDM4E$^{1–337}$, KDM5C$^{1–765}$, and KDM6B$^{1141–1,641}$. 31

Table S7 Summary of screen of histone and non-histone peptides with a panel of KDMs for RDM activity. 32

Table S8 Summary of results with unmodified, methylated, and citrullinated histone H3 and H4 peptides from the histone peptide library (AltaBiosciences Set 5) screened with KDM4A, KDM4D, KDM4E, and KDM5C. 33

Table S9 Comparison of sequences surrounding potential arginine substrates of the KDMs. 34

References 35

(A)

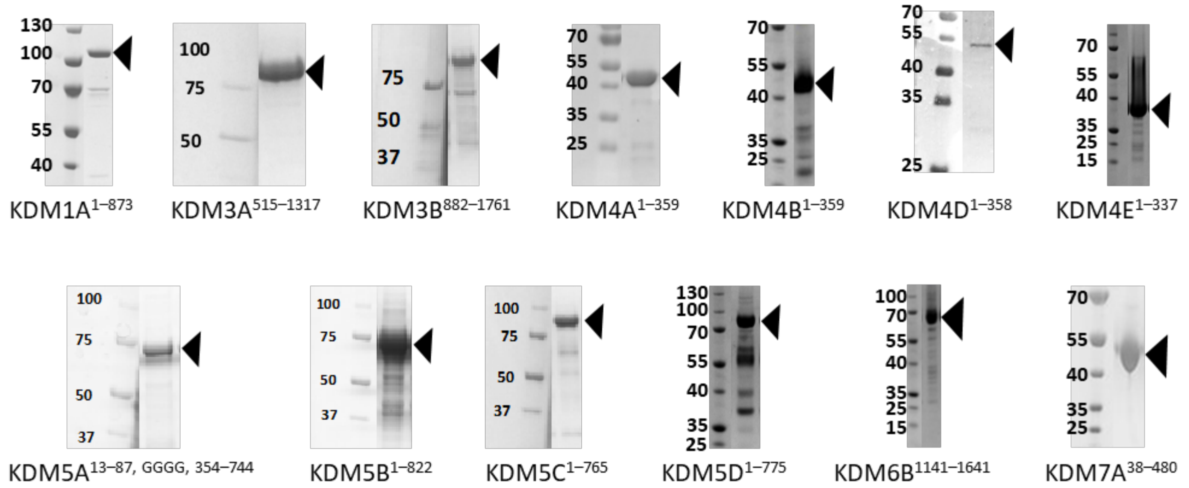

(B)

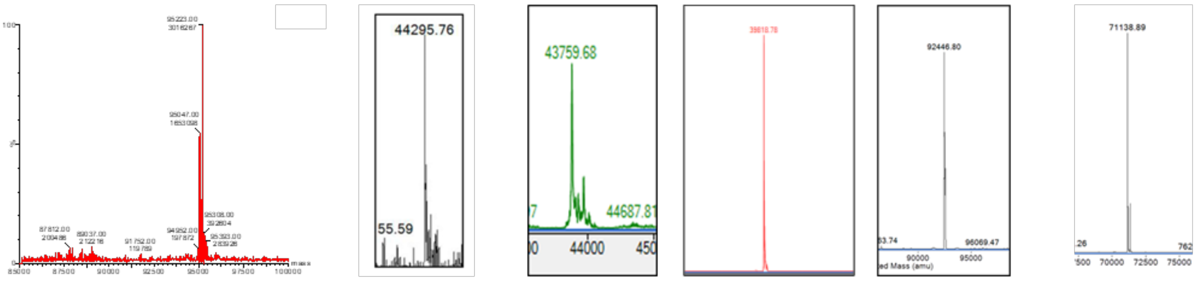

Enzyme	$KDM1A^{1–873}$	$KDM4B^{1–359}$	$KDM4D^{1–358}$	$KDM4E^{1–337}$	$KDM5D^{1–775}$	$KDM6B^{1141–1641}$
Calculated MW (Da)	95204	44295	43752	39818	92407	71136
Observed MW (Da)	95223	44295	43760	39819	92447	71139

Figure S1 Characterisation of purified enzymes. (A) SDS-PAGE analysis demonstrating purities of enzymes used in our work. See **Table S1** for construct information. **(B)** Deconvoluted LC–MS analyses for selected enzymes.

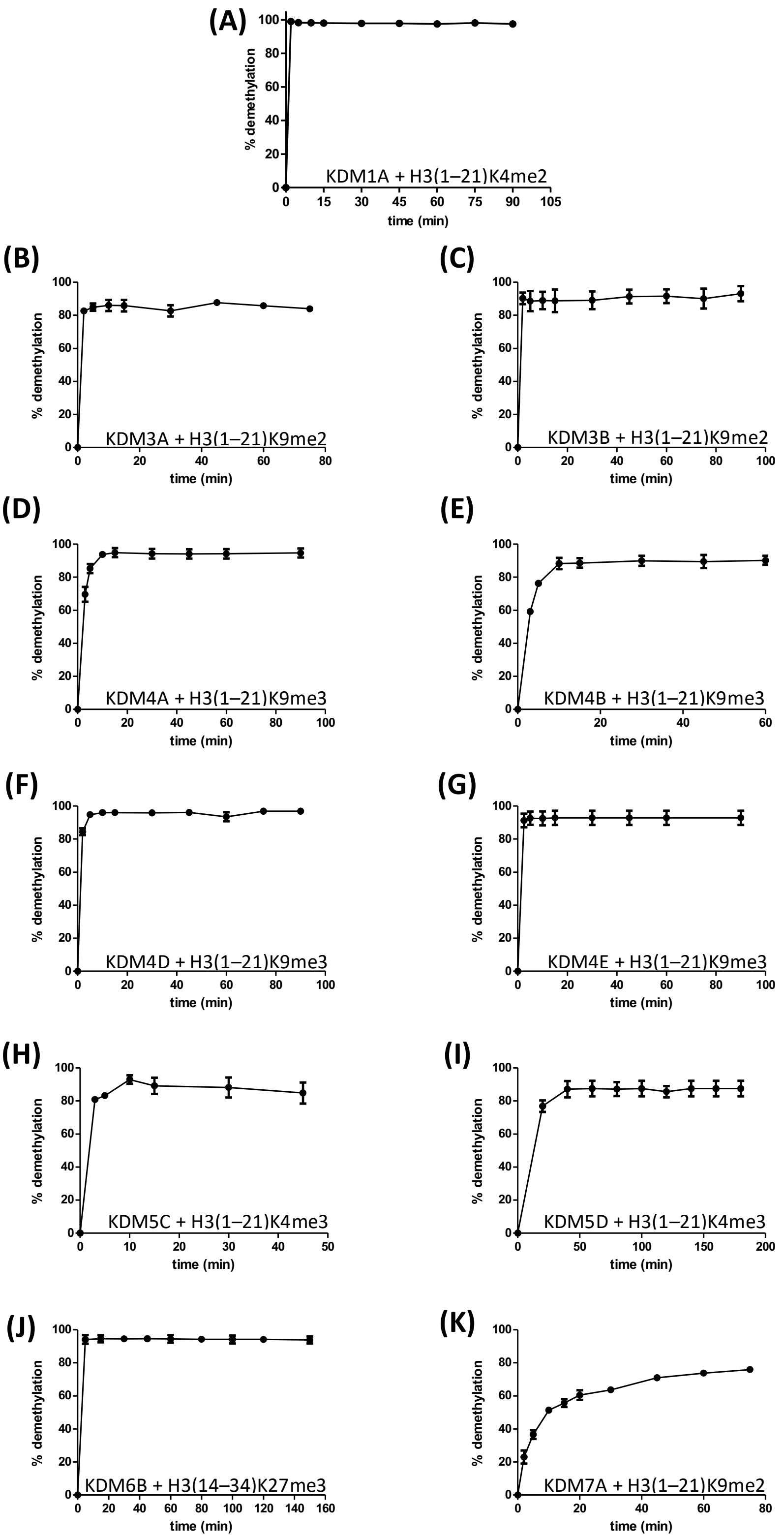
(A)
KDM1A + H3(1–21)K4me2
(B)
KDM3A + H3(1–21)K9me2
(C)
KDM3B + H3(1–21)K9me2
(D)
KDM4A + H3(1–21)K9me3
(E)
KDM4B + H3(1–21)K9me3
(F)
KDM4D + H3(1–21)K9me3
(G)
KDM4E + H3(1–21)K9me3
(H)
KDM5C + H3(1–21)K4me3
(I)
KDM5D + H3(1–21)K4me3
(J)
KDM6B + H3(14–34)K27me3
(K)
KDM7A + H3(1–21)K9me2
% demethylation
time (min)

(L)

Enzyme	Peptide	Time course	Approximate max peptide turnover (%)	Time (min)
KDM1A	H3(1–21)K4me2	A	100	2
KDM3A	H3(1–21)K9me2	B	85	5
KDM3B	H3(1–21)K9me2	C	90	2
KDM4A	H3(1–21)K9me3	D	94	10
KDM4B	H3(1–21)K9me3	E	90	10
KDM4D	H3(1–21)K9me3	F	96	5
KDM4E	H3(1–21)K9me3	G	92	5
KDM5C	H3(1–21)K4me3	H	93	10
KDM5D	H3(1–21)K4me3	I	87	40
KDM6B	H3(14–34)K27me3	J	94	5
KDM7A	H3(1–21)K9me2	K	76	75

Figure S2 Characterisation of enzyme activities with established substrates. Time courses showing percentage demethylation of established KDM substrates as analysed by MALDI–TOF MS. The demethylation of H3(1–21)K9me2 as catalysed by **(B)** KDM3A and **(C)** KDM3B; demethylation of H3(1–21)K9me3 as catalysed by **(D)** KDM4A, **(E)** KDM4B, **(F)** KDM4D, and **(G)** KDM4E; demethylation of H3(1–21)K4me3 as catalysed by **(H)** KDM5C and **(I)** KDM5D; demethylation of **(J)** H3(14–34)K27me3 as catalysed by KDM6B and of H3(1–21)K9me2 by **(A)** KDM1A and **(K)** KDM7A. Error bars: +/- stdev, n = 3 (technical triplicates). Conditions: see Table S3. The maximum percentage turnover of the respective peptides (10 µM) observed and time taken to reach this are given in **(L)**.

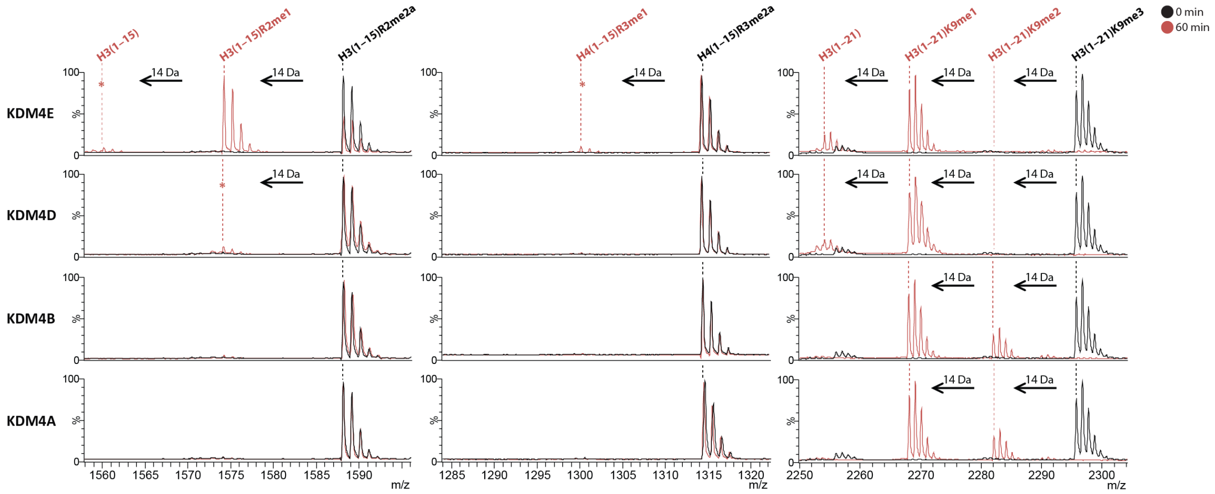

Figure S3 Initial screening of KDM4A/B/D/E with H3(1–15)R2me2a and H4(1–15)R3me2a. Representative MALDI–TOF MS spectra following 60-minute incubation of KDM4E, KDM4D, KDM4B, and KDM4A with (left) H3(1–15)R2me2a, (middle) H4(1–15)R3me2a, and (right) H3(1–21)K9me3 as a positive control. MALDI–TOF MS analyses reveals −14 Da peaks from H3(1–21)K9me3 (positive control) for all KDM4s and for KDM4E from H3(1–15)R2me2a and H4(1–15)R3me2a, and for KDM4D from H3(1–15)R2me2a are observed. No mass shifts were observed for KDM4B and KDM4A with H3(1–15)R2me2a and for KDM4D, KDM4B, and KDM4A with H4(1–15)R3me2a. (*) = low level (~10%) peaks in the MALDI–TOF MS indicating potential demethylation. Representative data are shown from n = 4 (independent assays) for positive controls; n = 2 (independent assays) for H3(1–15)R2me2a and H4(1–15)R3me2a. Charge state of labelled ions: $[MH]^+$. Y-axis: relative abundance (%). Conditions: 2 µM enzyme, 10 µM peptide, 100 µM 2OG, 100 µM sodium L-ascorbate, 10 µM $(NH_4)_2Fe(SO_4)_2$, and 50 mM HEPES (pH 7.5).

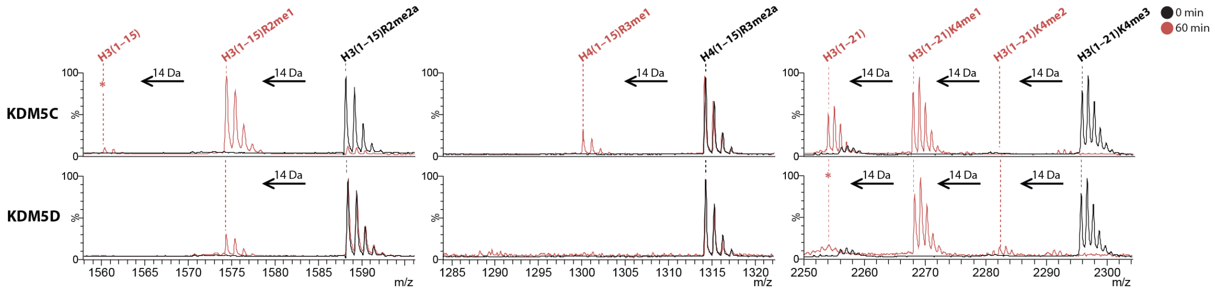

Figure S4 Initial screening of KDM5C/D with H3(1–15)R2me2a and H4(1–15)R3me2a. Representative MALDI–TOF MS following 60-minute incubation of KDM5C and KDM5D, with (left) H3(1–15)R2me2a peptide as a positive control, (middle) H4(1–15)R3me2a, and (right) H3(1–21)K3me3 showing −14 Da shifts in mass corresponding to removal of methyl groups in all traces except for KDM5D with H4(1–15)R3me2a. (*) = low level peaks in the MALDI–TOF MS indicating ~10% demethylation. Representative data are shown from n = 4 (independent assays) for positive controls; n = 2 (independent assays) for H3(1–15)R2me2a and H4(1–15)R3me2a. Charge state of labelled ions: $[MH]^+$. Y-axis: relative abundance (%). Conditions: 2 µM enzyme, 10 µM peptide, 100 µM 2OG, 100 µM sodium L-ascorbate, 10 µM $(NH_4)_2Fe(SO_4)_2$, and 50 mM HEPES (pH 7.5), and 5 mM TCEP and 50 mM NaCl for KDM5D.

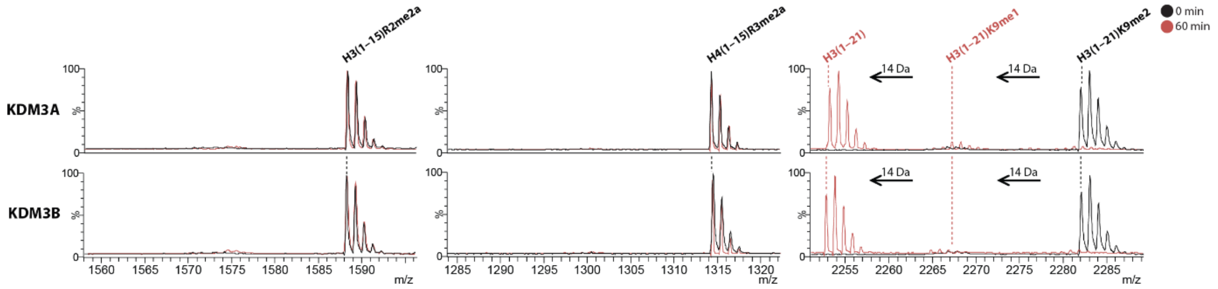

Figure S5 Initial screening of KDM3A/B with H3(1–15)R2me2a and H4(1–15)R3me2a. Representative MALDI–TOF MS following 60-minute incubation of (from top to bottom) KDM3A and KDM3B, with (left) H3(1–15)R2me2a, (middle) H4(1–15)R3me2a, and (right) H3(1–21)K9me2 peptide as a positive control. No mass shifts were observed for KDM3A and KDM3B with H3(1–15)R2me2a and with H4(1–15)R3me2a. Representative data are shown from n = 4 (independent assays) for positive controls; n = 2 (independent assays) for H3(1–15)R2me2a and H4(1–15)R3me2a. Charge state of labelled ions: $[MH]^+$. Y-axis: relative abundance (%). Conditions: 2 μM enzyme, 10 μM peptide, 100 μM 2OG, 100 μM sodium L-ascorbate, 50 μM $(NH_4)_2Fe(SO_4)_2$, 50 mM HEPES (pH 7.5), and 5 mM tris(2-carboxyethyl)phosphine (TCEP).

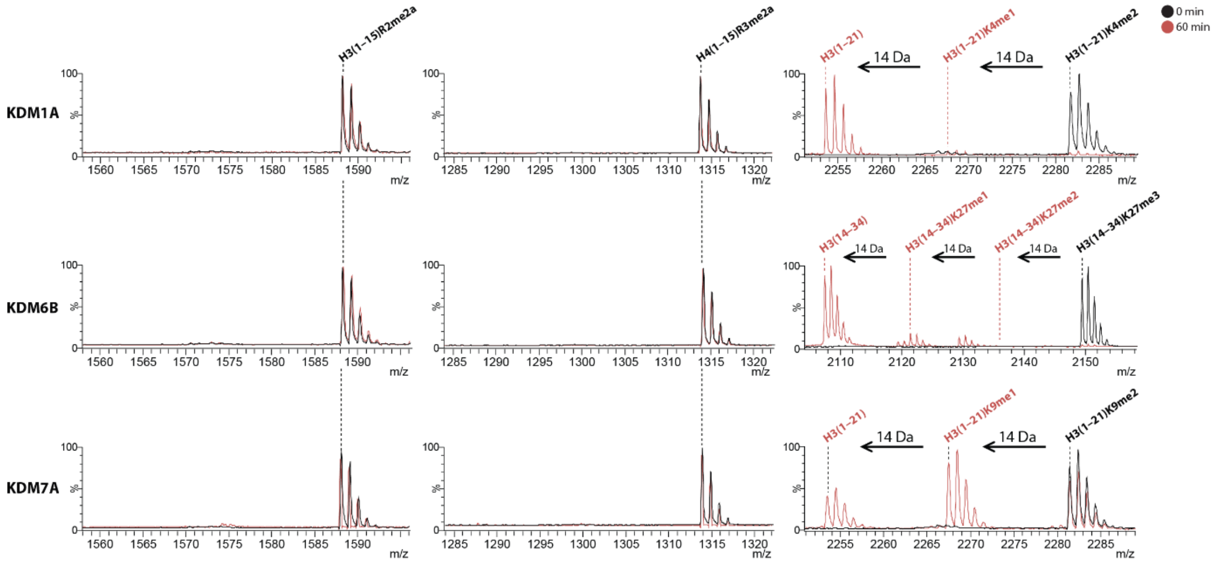

Figure S6 Initial screening of KDM1A, KDM6B and KDM7A with H3(1–15)R2me2a and H4(1–15)R3me2a. Representative MALDI–TOF MS following 60-minute incubation with KDM1A, KDM6Bm, and KDM7A (left) H3(1–15)R2me2a, (middle) H4(1–15)R3me2a showing no mass shifts, and (right) an H3 peptide as a positive control. **KDM1A** with H3(1–15)R2me2a, H4(1–15)R3me2a, and H3(1–21)K4me2; **KDM6B** with H3(1–15)R2me2a, H4(1–15)R3me2a, and H3(14–34)K27me3; and **KDM7A** with H3(1–15)R2me2a, H4(1–15)R3me2a, and H3(1–21)K9me2. Representative data shown from n = 4 (independent assays) for positive controls; n = 2 (independent assays) for H3(1–15)R2me2a and H4(1–15)R3me2a. Charge state of labelled ions: $[MH]^+$. Y-axis: relative abundance (%). **Conditions KDM1A:** 2 µM enzyme, 10 µM peptide, 20 mM Tris-HCl (pH 8.0), 0.01% Triton X-100, 4 mM TCEP, temp: 37°C, **KDM6B:** 2 µM enzyme, 10 µM peptide, 100 µM 2OG, 100 µM sodium L-ascorbate, 10 µM $(NH_4)_2Fe(SO_4)_2$, and 50 mM HEPES (pH 7.5), and 5 mM TCEP and 50 mM NaCl, **KDM7A:** 4 µM enzyme, 10 µM peptide, 100 µM 2OG, 200 µM sodium L-ascorbate, 50 µM $(NH_4)_2Fe(SO_4)$2, 50 mM HEPES (pH 7.5), 50 mM NaCl, 5 mM TCEP, 37 °C.

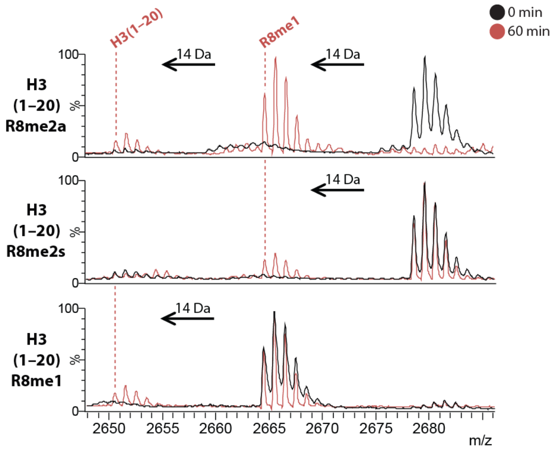

Figure S7 Representative MALDI–TOF MS following 60-minute incubation of KDM5C with H3R8 peptides (from top to bottom) H3(1–20)R8me2a, H3(1–20)R8me2s, and H3(1–20)R8me1 peptides from the AltaBioscience peptide library (Set 5). −14 Da shifts in mass from all peptides corresponding to removal of methyl groups were observed. Representative data are shown from n = 2 (independent assays). All peptides were linked (via an aminohexanoyl group) to N^{ε}-(D-biotin)-L-lysine-amide. Charge state of labelled ions: $[MH]^{+}$. Y-axis: relative abundance (%). Conditions: 2 µM KDM5C, 10 µM peptide, 100 µM 2OG, 100 µM sodium L-ascorbate, 10 µM $(NH_4)_2Fe(SO_4)_2$, and 50 mM HEPES (pH 7.5).

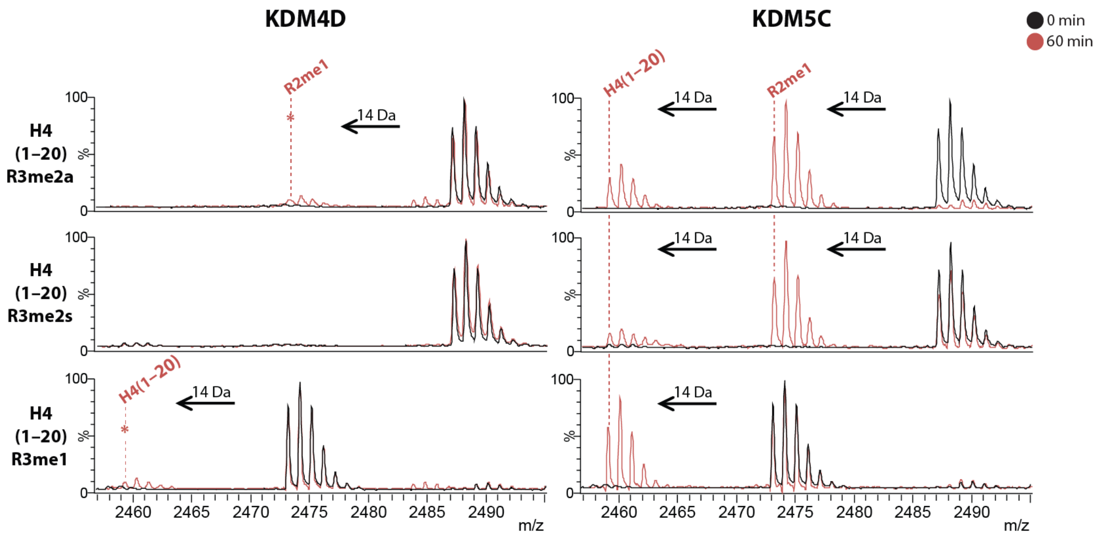

Figure S8 Representative MALDI–TOF MS following 60-minute incubation of (left) KDM4D and (right) KDM5C with H4R3 peptides (from top to bottom): H4(1–20)R3me2a, H4(1–20)R3me2s, and H4(1–20)R3me1 peptides from the AltaBioscience phosphorylation and arginine methylation histone library (Set 5). Low levels of −14 Da shifts from H4(1–20)R3me2a and H4(1–20)R3me1 corresponding to potential removal of a methyl group were observed with KDM4D; −14 Da shifts from H4(1–20)R3me2a, H4(1–20)R3me2s, and H4(1–20)R3me1 corresponding to removal of a methyl group were observed with KDM5C. No mass shifts were observed for KDM4D with H4(1–20)R3me2s. (*) = low level peaks in the MALDI–TOF MS indicating ~10% demethylation. Representative data shown from n = 2 (independent assays). All peptides were linked (via an aminohexanoyl group) to N^{ε}-(D-biotin)-L-lysine-amide. Charge state of labelled ions: $[MH]^+$. Y-axis: relative abundance (%). Conditions KDM4D assay: 2 µM enzyme, 10 µM peptide, 100 µM 2OG, 100 µM sodium L-ascorbate, 10 µM $(NH_4)_2Fe(SO_4)_2$, and 50 mM HEPES (pH 7.5), KDM5C assay: 2 µM enzyme, 10 µM peptide, 100 µM 2OG, 100 µM sodium L-ascorbate, 10 µM $(NH_4)_2Fe(SO_4)_2$, and 50 mM HEPES (pH 7.5).

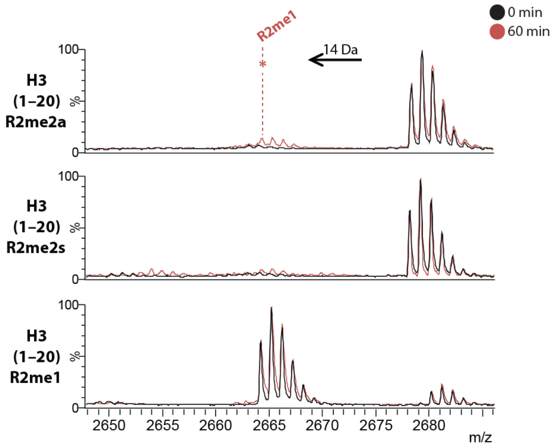

Figure S9 Representative MALDI–TOF MS following 60-minute incubation of KDM4D with H3R2 peptides (from top to bottom): H3(1–20)R2me2a, H3(1–20)R2me2s, and H3(1–20)R2me1 peptides from AltaBioscience peptide library (Set 5). −14 Da shifts in mass from H3(1–20)R2me2a indicating ~10% demethylation potentially corresponding to removal of a methyl group were observed. No mass shifts were observed with H3(1–20)R3me2s and H3(1–20)R2me1. (*) = low level peaks in the MALDI–TOF MS indicating ~10% demethylation. Representative data are shown from n = 2 (independent assays). All peptides were linked (via an aminohexanoyl group) to N^{ε}-(D-biotin)-L-lysine-amide. Charge state of labelled ions: $[MH]^+$. Y-axis: relative abundance (%). Conditions: 2 µM KDM4D, 10 µM peptide, 100 µM 2OG, 100 µM sodium L-ascorbate, 10 µM $(NH_4)_2Fe(SO_4)_2$, and 50 mM HEPES (pH 7.5).

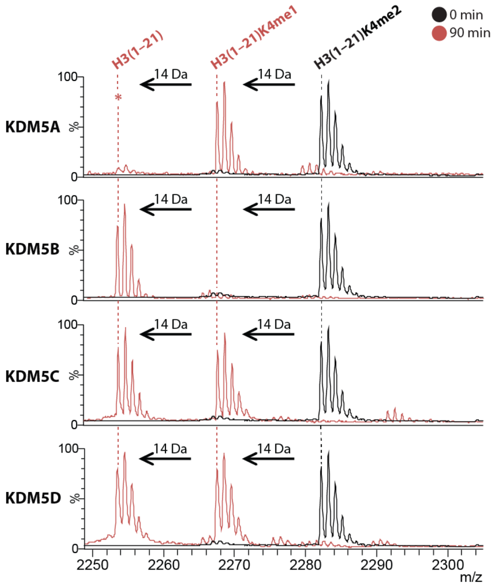

Figure S10 Positive control for experiment in Figure 2A (main text). Representative MALDI–TOF MS showing multiple -14 Da decreases in mass from H3(1–21)K4me3 corresponding to removal of methyl groups following incubation for 90 minutes with KDM5A, KDM5B, KDM5C, and KDM5D. Charge state of labelled ions: $[MH]^{+}$. (*) = low level peaks in the MALDI–TOF MS indicating ~10% demethylation. Y-axis: relative abundance (%). Conditions: 2 µM enzyme, 10 µM peptide, 10 µM $(NH_4)_2Fe(SO_4)_2$, 100 µM sodium L-ascorbate, 100 µM 2OG, 50 mM HEPES pH 7.5, 50 mM NaCl, 5 mM TCEP, and 37°C.

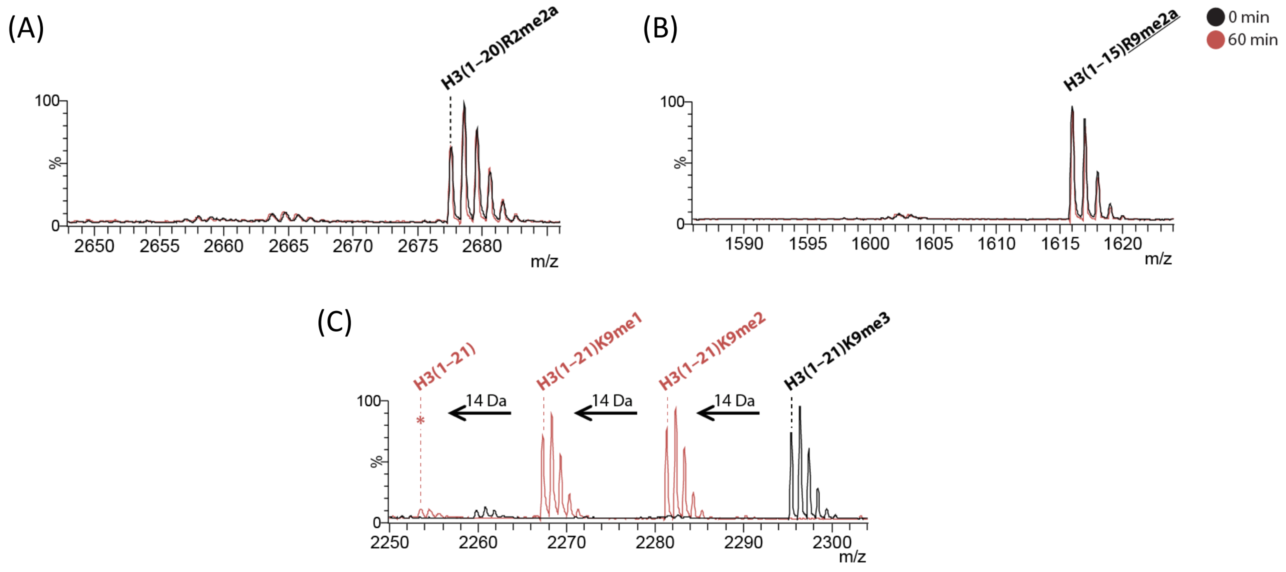

Figure S11 RDM activity was not observed with a higher concentration of KDM4A$^{1–359}$. Representative MALDI–TOF MS showing no mass shifts from **(A)** H3(1–20)R2me2a and **(B)** H3(1–15)R9me2a and showing −14 Da decreases in mass (corresponding to removal of methyl groups) from **(C)** H3(1–21)K9me3 following incubation for 60 minutes with KDM4A$^{1–359}$. (*) = low level peaks in the MALDI–TOF MS indicating ~10% demethylation. n = 3 (independent assays). H3(1–20)R2me2a linked via an aminohexanoyl group to N^{ε}-(D-biotin)-L-lysine-amide. Charge state of labelled ions: $[MH]^{+}$. Y-axis: relative abundance (%). Conditions: 4 µM KDM4A, 10 µM peptide, 100 µM 2OG, 100 µM sodium L-ascorbate, 10 µM $(NH_4)_2Fe(SO_4)_2$, and 50 mM HEPES (pH 7.5).

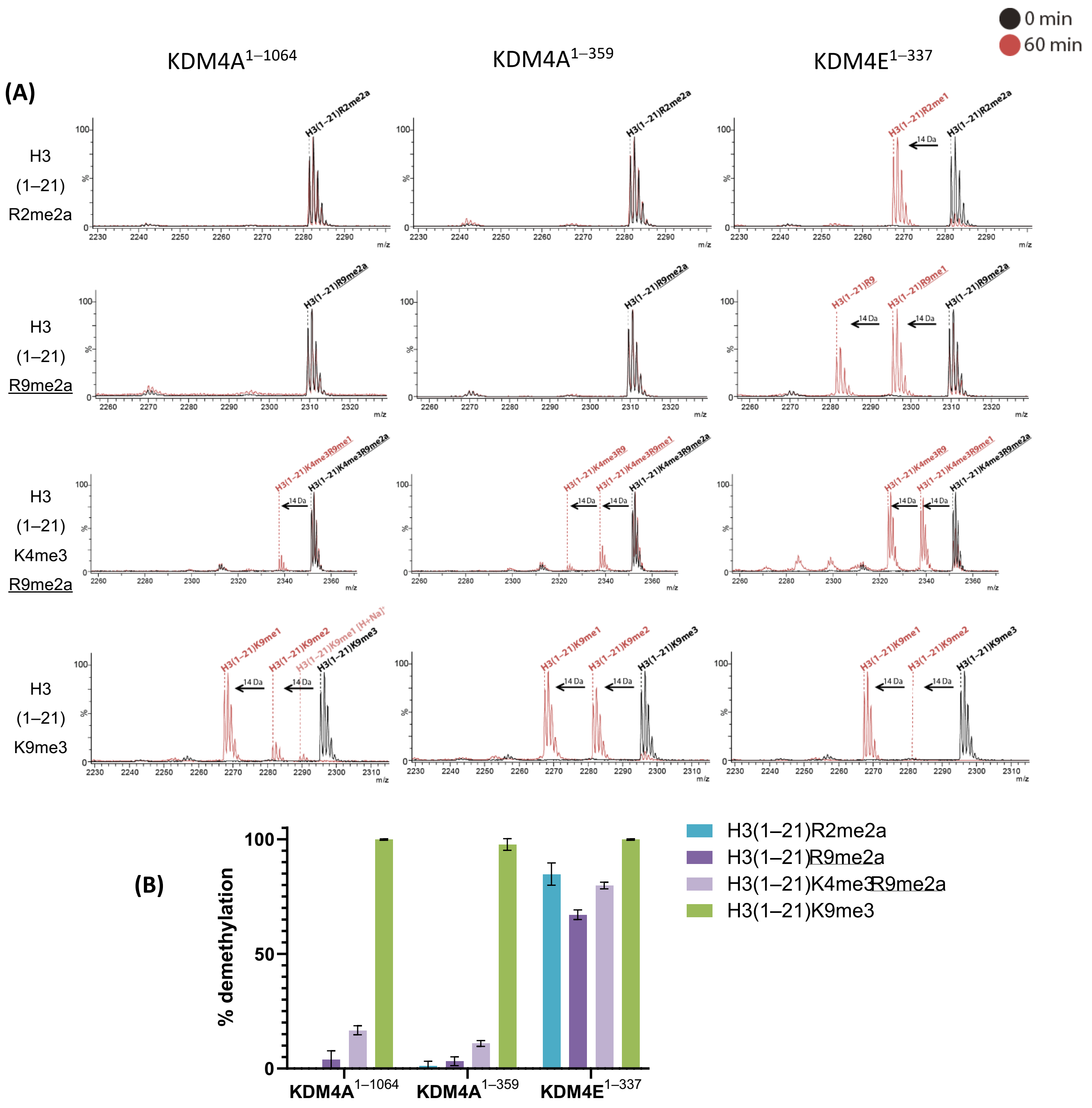

Figure S12 KDM4A$^{1–1064}$ and KDM4A$^{1–359}$ have similar RDM activities. (A) Representative MALDI–TOF MS following 60-minute incubation of KDM4A$^{1–1064}$ (left), KDM4A$^{1–359}$ and KDM4E$^{1–337}$ (right) with (from top to bottom) H3(1–21)R2me2a, H3(1–21)R9me2a, H3(1–21)K4me3R9me2a, and H3(1–21)K9me3 showing -14 Da decreases in mass from the peptides corresponding to removal of methyl groups; Representative data are shown from n = 3 (independent assays). Charge state of labelled ions: $[MH]^+$. Y-axis: relative abundance (%). **(B)** Bar graph comparing % demethylation of KDM4A$^{1–1064}$, KDM4A$^{1–359}$ and KDM4E$^{1–337}$ of H3(1–21)R2me2a, H3(1–21)R9me2a, H3(1–21)K4me3R9me2a, and H3(1–21)K9me3, analysed by MALDI–TOF MS. Error bars represent +/- stdev; n = 3 (independent assays). Conditions: 1 µM KDM4A$^{1–359}$ and KDM4E$^{1–337}$, 10 µM peptide, 100 µM 2OG, 100 µM sodium L-ascorbate, 10 µM $(NH_4)_2Fe(SO_4)_2$, and 50 mM HEPES (pH 7.5); 1 µM KDM4A$^{1–1064}$, 10 µM peptide 100 µM 2OG, 100 µM sodium L-ascorbate, 50 µM $(NH_4)_2Fe(SO_4)_2$, and 50 mM HEPES (pH 7.5), 1 mM TCEP, 0.02% (v/v) Triton-X.

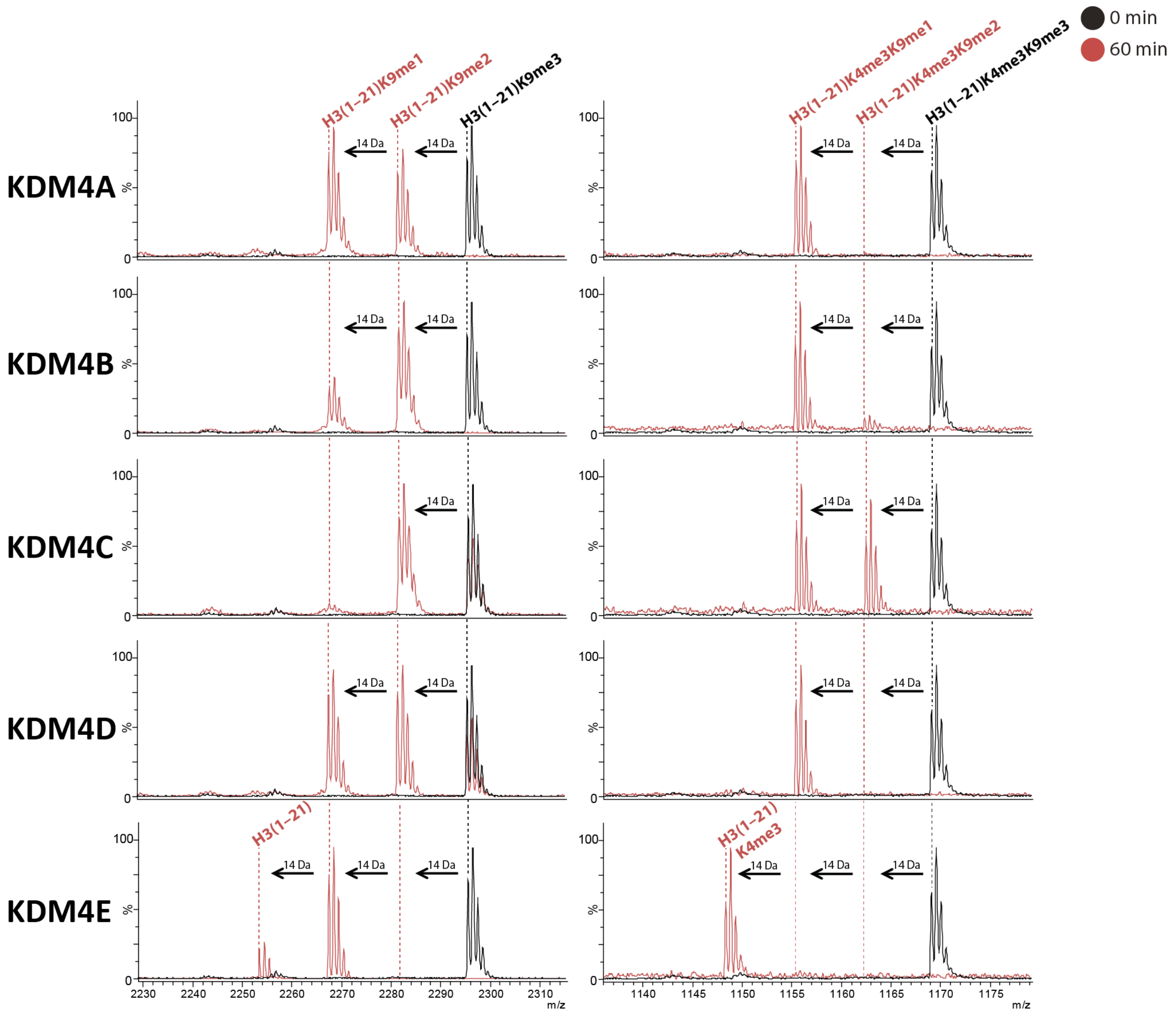

Figure S13A KDM4s and H3(1–21)K9me3 and H3(1–21)K4me3K9me3. Representative MALDI–TOF MS following 60-minute incubations of KDM4A, KDM4B, KDM4C, KDM4D and KDM4E showing -14 Da decreases in mass from (left) H3(1–21)K9me3 and (right) H3(1–21)K4me3K9me3 corresponding to removal of methyl groups. Representative data are shown from n = 3 (independent assays). Charge state of labelled ions: left: $[MH]^{+}$, right: $[M + H]^{2+}$. Y-axis: relative abundance (%). See Table S3 for assay conditions.

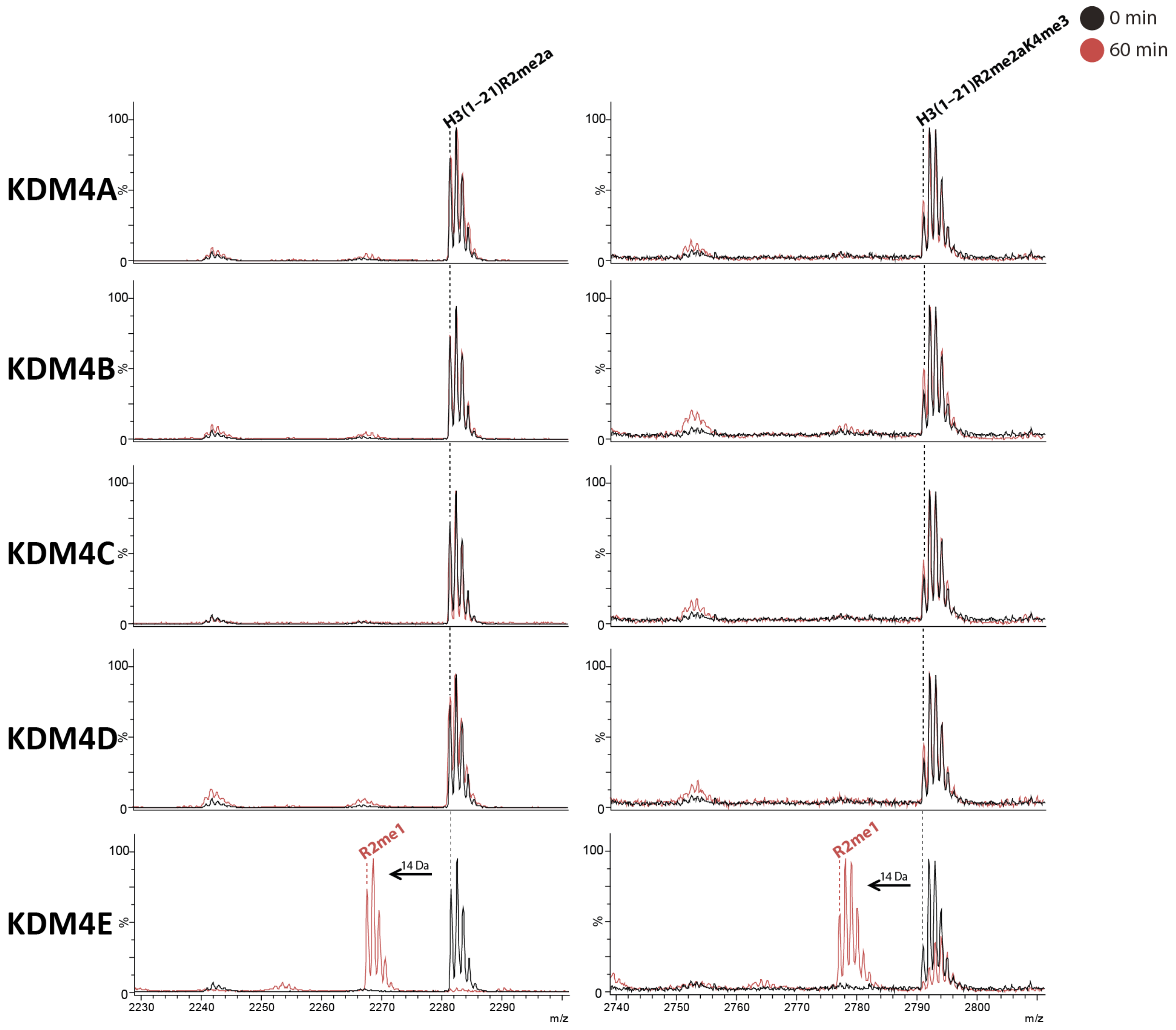

Figure S13B KDM4s and H3(1–21)R2me2a and H3(1–21)R2me2aK4me3. Representative MALDI–TOF MS following 60-minute incubation of KDM4A, KDM4B, KDM4C, KDM4D and KDM4E showing -14 Da decreases in mass from (left) H3(1–21)R2me2a and (right) H3(1–21)R2me2aK4me3 corresponding to removal of methyl groups with KDM4E but not with KDM4A–D; Representative data shown from n = 3 (independent assays). Charge state of labelled ions: $[MH]^+$. Y-axis: relative abundance (%). H3(1–20)R2me2aK4me3 was linked via an aminohexanoyl group to N^{ε}-(D-biotin)-L-lysine-amide. See Table S3 for assay conditions.

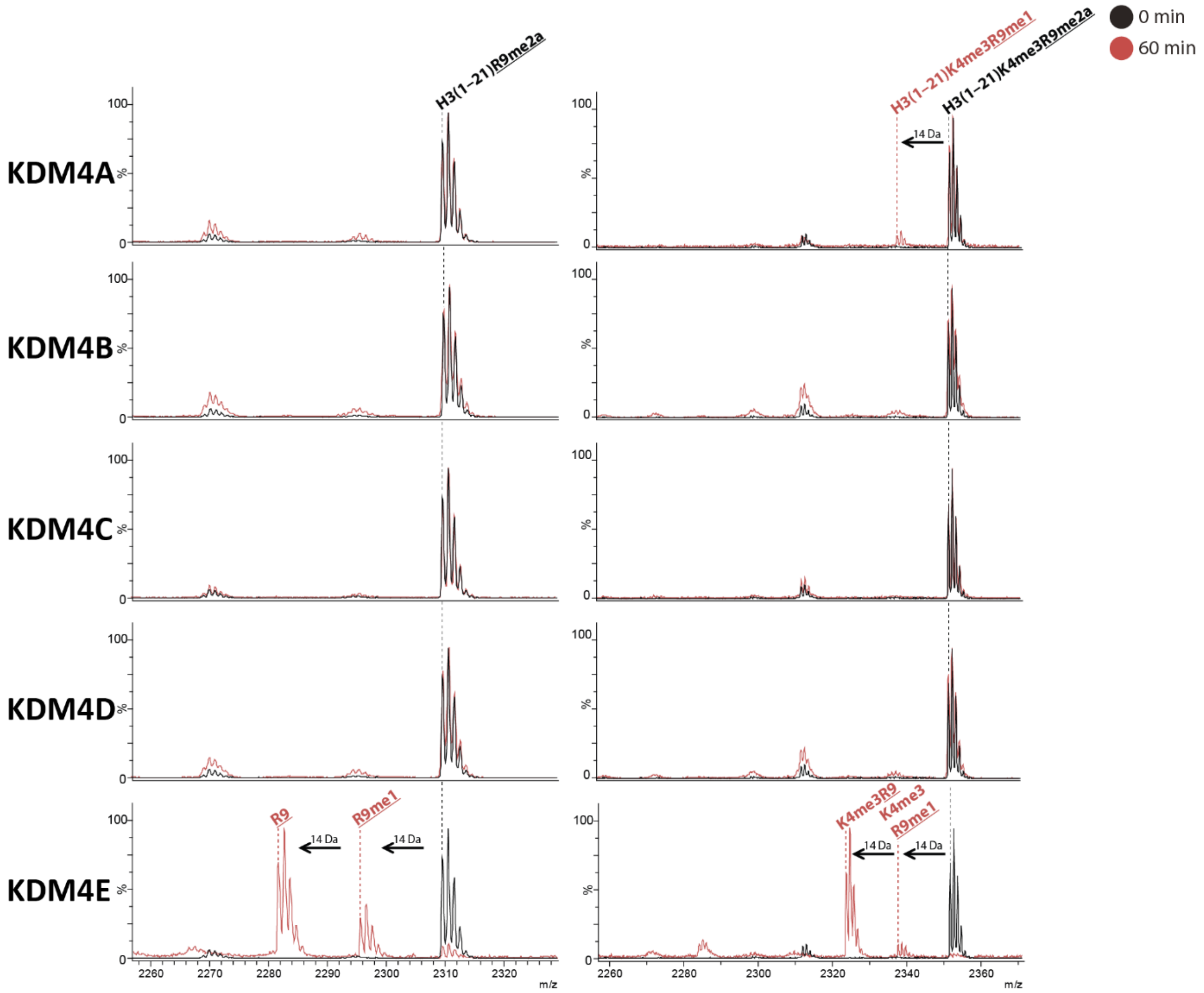

Figure S13C KDM4s and H3(1–21)R9me2a and H3(1–21)K4me3R9me2a. Representative MALDI–TOF MS following 60-minute incubations of KDM4A, KDM4B, KDM4C, KDM4D and KDM4E showing −14 Da decreases in mass from (left) H3(1–21)R9me2a and (right) H3(1–21)K4me3R9me2a corresponding to removal of methyl groups with KDM4E for both peptides and with H3(1–21)K4me3R9me2a and KDM4A; Representative data are shown from n = 3 (independent assays). Charge state of labelled ions: $[MH]^{+}$. Y-axis: relative abundance (%). See Table S3 for assay conditions.

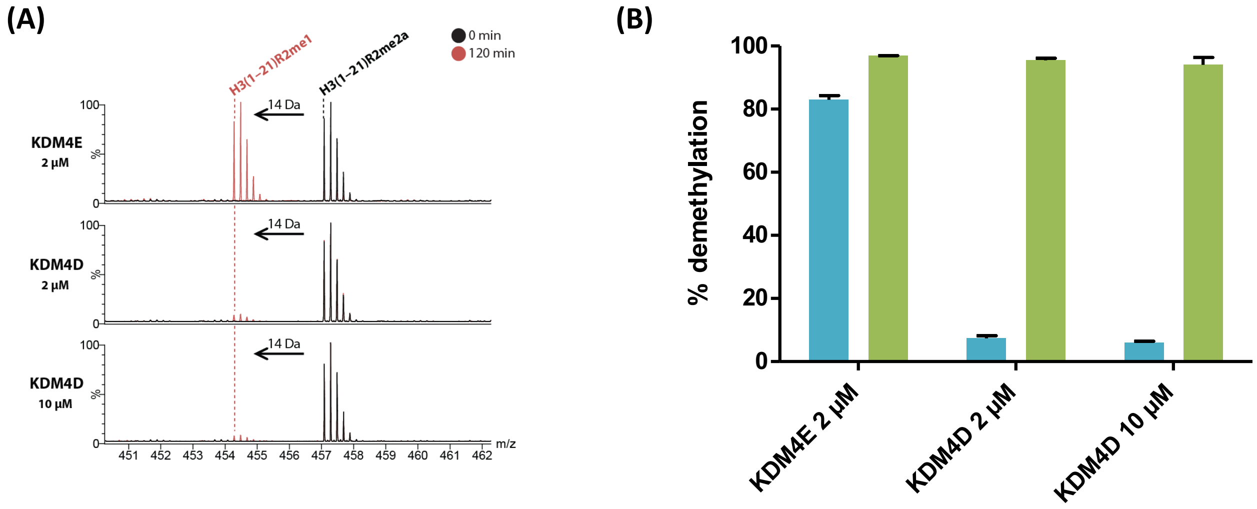

(C)

Enzyme	Concentration (µM)	% demethylation ± stdev	
		H3(1–21)R2me2a	H3(1–21)K9me3
KDM4E	2	83 ± 1.83	97 ± 0.05
KDM4D	2	7 ± 1.17	95 ± 0.53
	10	6 ± 0.79	94 ± 1.91

Figure S14 Comparison of RDM activities of KDM4E and KDM4D with H3(1–21)R2me2a. (A) Representative LC–MS showing 120-minute incubation of (from top to bottom) KDM4E at 2 µM, KDM4D at 2 µM, and KDM4D at 10 µM showing −14 Da decreases in mass from H3(1–21)R2me2a corresponding to removal of methyl groups. Charge state of labelled ions: $[M + H]^{5+}$. Y-axis: relative abundance (%). **(B)** Bar graph comparing the % demethylation of KDM4D and KDM4E of H3(1–21)R2me2a (in **cyan)** and H3(1–21)K9me3 (in **green)**, analysed by LC–MS. Error bars represent +/- stdev; n = 3 (independent assays). **(C)** Table comparing the % demethylation of KDM4D and KDM4E of H3(1–21)R2me2a and H3(1–21)K9me3, analysed by LC–MS. n = 3 (independent assays). Conditions: 10 µM peptide, 200 µM 2OG, 100 µM sodium L-ascorbate, 10 µM $(NH_4)_2Fe(SO_4)_2$, and 50 mM HEPES (pH 7.5).

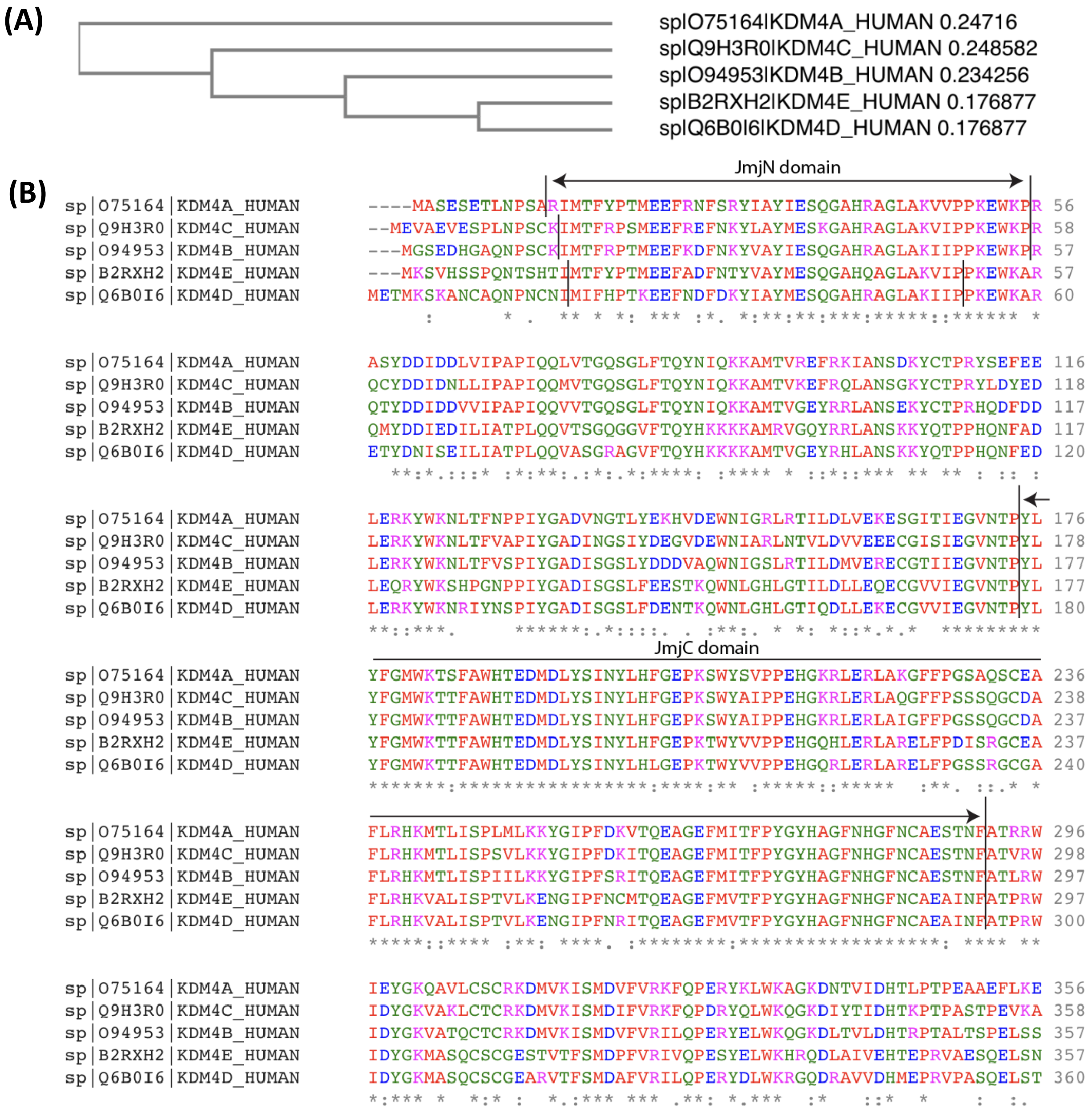

Figure S15 Analysis of the KDM4 sequences using Clustal omega. (A) Alignment of human KDM4A, KDM4B, KDM4C, KDM4D, and KDM4E. **(B)** Sequence alignment of the JmjN and JmjC domains (marked with arrows) of the KDM4 proteins.

(A)

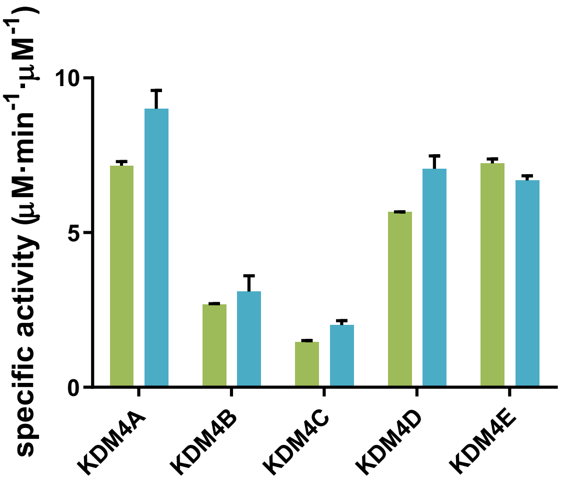

(B)

Enzyme	Specific activity ± stdev ($\mu M \cdot min^{-1} \cdot \mu M^{-1}$)	
	**H3(1–15)K9me3**	**H3(1–21)K9me3**
KDM4A	7.16 ± 0.19	9.01 ± 0.48
KDM4B	2.67 ± 0.05	3.09 ± 0.42
KDM4C	1.46 ± 0.08	2.01 ± 0.12
KDM4D	5.66 ± 0.01	7.06 ± 0.34
KDM4E	7.24 ± 0.20	6.69 ± 0.12

Figure S16 Comparison of demethylation of H3(1–15)K9me3 (green) and H3(1–21)K9me3 (blue) as catalysed by 1 µM of KDM4A, KDM4B, KDM4C, KDM4D, and KDM4E. Assays were over 60 minutes with measurement using the FDH assay. Results are represented as a **(A)** bar chart and **(B)** a table. Error bars represent +/- stdev; n = 3 (independent assays). Conditions: 100 µM peptide, 10 µM $(NH_4)_2Fe(SO_4)_2$, 100 µM sodium L-ascorbate, 200 µM 2OG, 50 mM HEPES pH 7.5, 0.01% (v/v) Tween, 500 µM β-NAD (β-nicotinamide adenine dinucleotide hydrate), and 1 µM FDH.

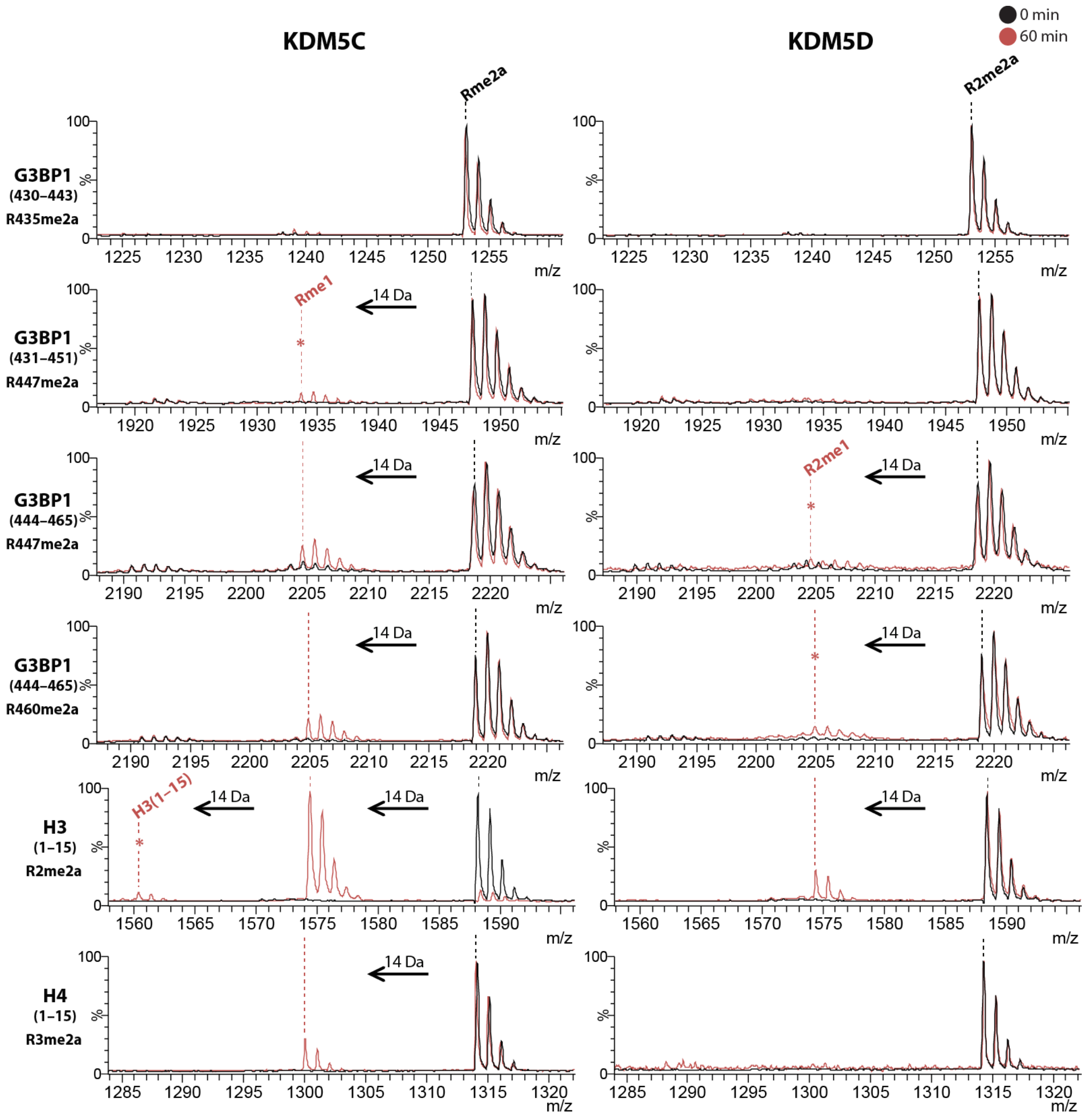

Figure S17 G3BP1 peptides with KDM5C and KDM5D. Representative MALDI–TOF MS following 60-minute incubations of KDM5C **(left)** and KDM5D **(right)** with (in order from top to bottom) G3BP1(430–443)R435me2a, G3BP1(431–451)R447me2a, G3BP1(444–465)R447me2a, and G3BP1(444–465)R460me2a showing −14 Da decreases in mass from the peptides corresponding to removal of methyl groups; representative data shown from n = 2 (independent assays). (*) = low level peaks in the MALDI–TOF MS indicating ~10% demethylation. Charge state of labelled ions: $[MH]^{+}$. Y-axis: relative abundance (%). Conditions: 2 µM enzyme, 10 µM peptide, 100 µM 2OG, 100 µM sodium L-ascorbate, 10 µM $(NH_4)_2Fe(SO_4)_2$, and 50 mM HEPES (pH 7.5), 5 mM TCEP, and 50 mM NaCl for KDM5D.

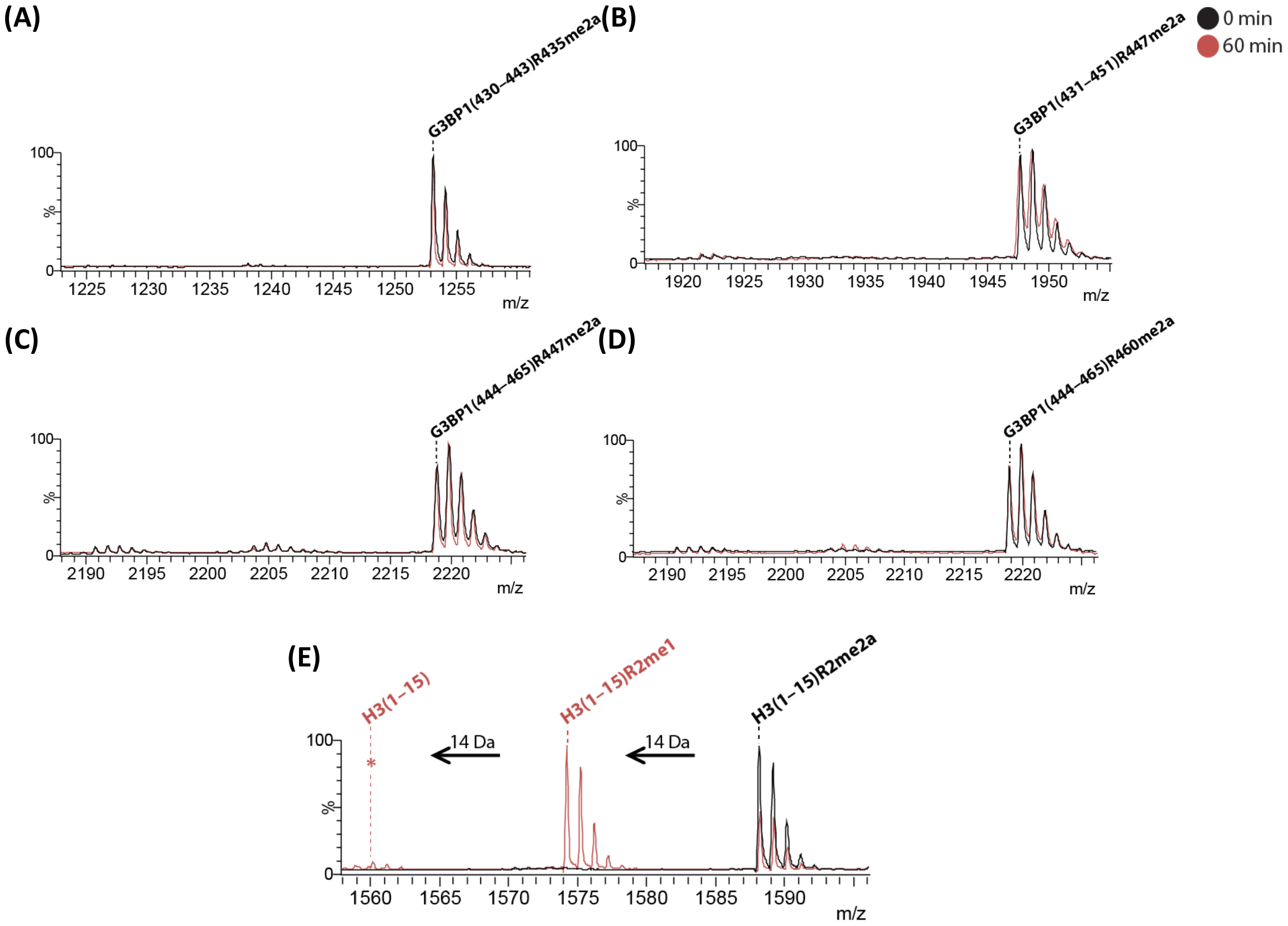

Figure S18 G3BP1 peptides with KDM4E. Representative MALDI–TOF MS following 60-minute incubations of KDM4E with **(A)** G3BP1(430–443)R435me2a, **(B)** G3BP1(431–451)R447me2a, **(C)** G3BP1(444–465)R447me2a, and **(D)** G3BP1(444–465)R460me2a peptides, showing no mass shifts, and **(E)** showing −14 Da decreases in mass from H3(1–15)R2me2a as the positive control, corresponding to removal of methyl groups. Representative data shown from n = 2 (independent assays). (*) = low level peaks in the MALDI–TOF MS indicating ~10% demethylation. Charge state of labelled ions: $[MH]^+$. Y-axis: relative abundance (%). Conditions: 2 µM KDM4E, 10 µM peptide, 100 µM 2OG, 100 µM sodium L-ascorbate, 10 µM $(NH_4)_2Fe(SO_4)_2$, and 50 mM HEPES (pH 7.5).

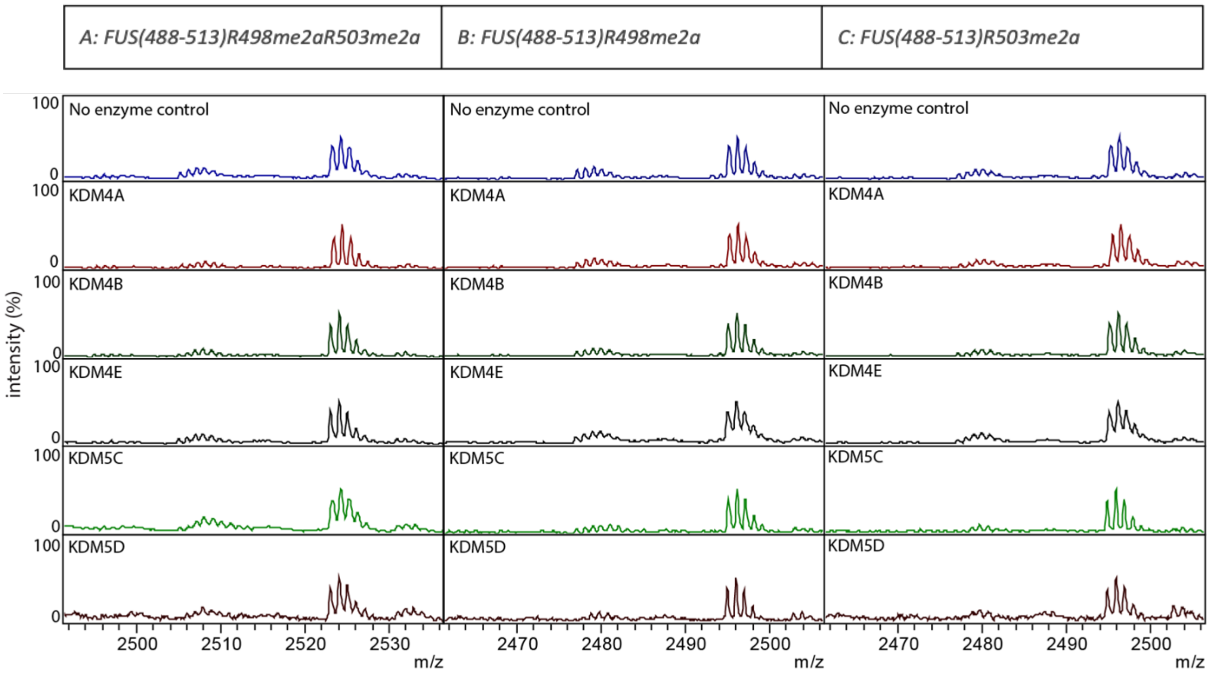

Figure S19 FUS peptides with KDM4A, KDM4B, KDM4E, KDM5C and KDM5D. Representative MALDI-TOF MS showing no shift in mass (i.e. no demethylation) after 60 minute incubation of **A:** FUS(488-513)R498me2aR503me2a, **B:** FUS(488-513)R498me2a, **C:** FUS(488-513)R503me2a by a selection of the enzymes tested (KDM4A, KDM4B, KDM4E, KDM5C and KDM5D). See Table S3 for assay conditions.

Enzyme	Sequence	Expressed in	Reference
KDM1A	1–873	*E. coli*	(1)
KDM3A	515–1317	Sf9 cells	(2, 3)
KDM3B	882–1761		
KDM4A*	1–1,064	Sf9 cells	
KDM4A	1–359	*E. coli*	(4)
KDM4B	1–359		(5)
KDM4C	1–359		
KDM4D	1–358		
KDM4E	1–337		
KDM5A	13–87, GGGG, 354–744	Sf9 cells	(6)
KDM5B	1–822		(7)
KDM5C	1–765		(2)
KDM5D	1–775		(8)
KDM6B	1141–1641	*E. coli*	(2)
KDM7A	38–480		(9, 10)
FDH (*Pseudomonas*)	1-399		(11)

Table S1 Summary of constructs and expression systems used. *KDM4A$^{1–1,064}$ was from Active Motif (Catalogue No: 31457).

Peptide	Sequence	Monoisotopic mass (Da)	$[M + H]^+$	$[M + H]^{5+}$	Source
H3(1–21)K4me3	ARTK[me3]QTARKSTGGKAPRKQLA	2294.38	2295.38	459.88	GL Biochem
H3(1–21)K9me2	ARTKQTARK[me2]STGGKAPRKQLA	2280.36	2281.36	457.07	GL Biochem
H3(1–21)K9me3	ARTKQTARK[me3]STGGKAPRKQLA-NH2	2294.38	2295.38	459.88	GL Biochem
H3(14–34)K27me3	KAPRKQLATKAARK[me3]SAPATGG	2148.3	2149.30	430.66	In house
H3(1–15)R2me2a	AR[me2a]TKQTARKSTGGKA	1586.93	1587.93	318.39	In house
H4(1–15)R3me2a	SGR[me2a]GKGGKGLGKGGA	1312.77	1313.77	263.55	In house
G3BP1(430–443)R435me2a	GPGGPR[me2a]GGLGGGMR	1251.66	1252.66	251.33	In house
G3BP1(431–451)R447me2a	PGGPRGGLGGGMRGPPR[me2a]GGMV	1946.02	1947.02	390.20	In house
G3BP1(444–465)R447me2a	GPPR[me2a]GGMVQKPGFGVGRGLAPR	2217.24	2218.24	444.45	In house
G3BP1(444–465)R460me2a	GPPRGGMVQKPGFGVGR[me2a]GLAPR	2217.24	2218.24	444.45	In house
FUS(488-513)R498me2aR503me2a	GGDRGGFRGGR[me2a]GGGDR[me2a]GGFGPGKMDS	2521.21	2522.21	505.24	In house
FUS(488-513)R498me2a	GGDRGGFRGGR[me2a]GGGDRGGFGPGKMDS	2493.18	2494.18	499.64	In house
FUS(488-513)R503me2a	GGDRGGFRGGRGGGDR[me2a]GGFGPGKMDS	2493.18	2494.18	499.64	In house
H3(1–20)(AB)	ARTKQTARKSTGGKAPRKQL -NH-Ahx-K-Biotin	2648.97	2649.97	530.79	AltaBioscience
H3(1–20)R2me1(AB)	AR[me1]TKQTARKSTGGKAPRKQL-NH-Ahx-K-Biotin	2662.98	2663.98	533.60	AltaBioscience
H3(1–20)R2me2s(AB)	AR[me2s]TKQTARKSTGGKAPRKQL-NH-Ahx-K-Biotin	2677.00	2678.00	536.40	AltaBioscience
H3(1–20)R2me2a(AB)	AR[me2a]TKQTARKSTGGKAPRKQL-NH-Ahx-K-Biotin	2677.00	2678.00	536.40	AltaBioscience
H3(1–21)R2me2aK4me3 (AB)	Biotin-K-Ahx-AR[me2a]TK[me3]QTARKSTGGKAPRKQLA	2790.08	2791.08	559.02	AltaBioscience
H3(1–20)R8me(AB)	ARTKQTAR[me1]KSTGGKAPRKQL-NH-Ahx-K-Biotin	2662.98	2663.98	533.60	AltaBioscience
H3(1–20)R8me2s(AB)	ARTKQTAR[me2s]KSTGGKAPRKQL-NH-Ahx-K-Biotin	2677.00	2678.00	536.40	AltaBioscience
H3(1–20)R8me2a(AB)	ARTKQTAR[me2a]KSTGGKAPRKQL-NH-Ahx-K-Biotin	2677.00	2678.00	536.40	AltaBioscience
H3(1–20)Ci8(AB)	ARTKQTAR[Ci]KSTGGKAPRKQL-NH-Ahx-K-Biotin	2553.87	2554.87	511.77	AltaBioscience
H3(1–20)R2me1R8me1(AB)	AR[me1]TKQTAR[me1]KSTGGKAPRKQL-NH-Ahx-K-Biotin	2677.00	2678.00	536.40	AltaBioscience
H3(1–20)R2me1R8me2s(AB)	AR[me1]TKQTAR[me2s]KSTGGKAPRKQL-NH-Ahx-K-Biotin	2594.93	2595.93	519.99	AltaBioscience
H3(1–20)R2me1R8me2a(AB)	AR[me1]TKQTAR[me2a]KSTGGKAPRKQL-NH-Ahx-K-Biotin	2594.93	2595.93	519.99	AltaBioscience
H3(1–20)R2me1Ci8(AB)	AR[me1]TKQTAR[Ci]KSTGGKAPRKQL-NH-Ahx-K-Biotin	2567.88	2568.88	514.58	AltaBioscience
H3(1–20)R2me2sR8me1(AB)	AR[me2s]TKQTAR[me1]KSTGGKAPRKQL-NH-Ahx-K-Biotin	2594.93	2595.93	519.99	AltaBioscience
H3(1–20)R2me2sR8me2s(AB)	AR[me2s]TKQTAR[me2s]KSTGGKAPRKQL-NH-Ahx-K-Biotin	2608.95	2609.95	522.79	AltaBioscience
H3(1–20)R2me2sR8me2a(AB)	AR[me2s]TKQTAR[me2a]KSTGGKAPRKQL-NH-Ahx-K-Biotin	2608.95	2609.95	522.79	AltaBioscience
H3(1–20)R2me2sCi8(AB)	AR[me2s]TKQTAR[Ci]KSTGGKAPRKQL-NH-Ahx-K-Biotin	2114.23	2115.23	423.85	AltaBioscience
H3(1–20)R2me2aR8me1(AB)	AR[me2a]TKQTAR[me1]KSTGGKAPRKQL-NH-Ahx-K-Biotin	2594.93	2595.93	519.99	AltaBioscience
H3(1–20)R2me2aR8me2s(AB)	AR[me2a]TKQTAR[me2s]KSTGGKAPRKQL-NH-Ahx-K-Biotin	2608.95	2609.95	522.79	AltaBioscience
H3(1–20)R2me2aR8me2a(AB)	AR[me2a]TKQTAR[me2a]KSTGGKAPRKQL-NH-Ahx-K-Biotin	2608.95	2609.95	522.79	AltaBioscience
H3(1–20)R2me2aCi8(AB)	AR[me2a]TKQTAR[Ci]KSTGGKAPRKQL-NH-Ahx-K-Biotin	2114.23	2115.23	423.85	AltaBioscience
H3(13–32)(AB)	Ac-GKAPRKQLATKAARKSAPAT-NH-Ahx-K-Biotin	2558.91	2559.91	512.78	AltaBioscience
H3(13–32)R17me1(AB)	Ac-GKAPR[me1]KQLATKAARKSAPAT-NH-Ahx-K-Biotin	2572.93	2573.93	515.59	AltaBioscience
H3(13–32)R17me2s(AB)	Ac-GKAPR[me2s]KQLATKAARKSAPAT-NH-Ahx-K-Biotin	2586.94	2587.94	518.39	AltaBioscience
H3(13–32)R17me2a(AB)	Ac-GKAPR[me2a]KQLATKAARKSAPAT-NH-Ahx-K-Biotin	2586.94	2587.94	518.39	AltaBioscience
H3(13–32)Ci17(AB)	Ac-GKAPR[Ci]KQLATKAARKSAPAT-NH-Ahx-K-Biotin	2559.90	2560.90	512.98	AltaBioscience
H3(13–32)R26me1(AB)	Ac-GKAPRKQLATKAAR[me1]KSAPAT-NH-Ahx-K-Biotin	2572.93	2573.93	515.59	AltaBioscience
H3(13–32)R26me2s(AB)	Ac-GKAPRKQLATKAAR[me2s]KSAPAT-NH-Ahx-K-Biotin	2586.94	2587.94	518.39	AltaBioscience
H3(13–32)R26me2a(AB)	Ac-GKAPRKQLATKAAR[me2a]KSAPAT-NH-Ahx-K-Biotin	2586.94	2587.94	518.39	AltaBioscience
H3(13–32)Ci26(AB)	Ac-GKAPRKQLATKAAR[Ci]KSAPAT-NH-Ahx-K-Biotin	2559.90	2560.90	512.98	AltaBioscience
H3(13–32)R17me2sR26me2s(AB)	Ac-GKAPR[me2s]KQLATKAAR[me2s]KSAPAT-NH-Ahx-K-Biotin	2614.97	2615.97	523.99	AltaBioscience
H3(13–32)R17me2sR26me2a(AB)	Ac-GKAPR[me2s]KQLATKAAR[me2a]KSAPAT-NH-Ahx-lysine-Biotin	2614.97	2615.97	523.99	AltaBioscience
H3(13–32)R17me2sCi26(AB)	Ac-GKAPR[me2s]KQLATKAAR[Ci]KSAPAT-NH-Ahx-lysine-Biotin	2587.93	2588.93	518.59	AltaBioscience
H3(13–32)R17me2aR26me2s(AB)	Ac-GKAPR[me2a]KQLATKAAR[me2s]KSAPAT-NH-Ahx-lysine-Biotin	2614.97	2615.97	523.99	AltaBioscience
H3(13–32)R17me2aR26me2a(AB)	Ac-GKAPR[me2a]KQLATKAAR[me2a]KSAPAT-NH-Ahx-lysine-Biotin	2614.97	2615.97	523.99	AltaBioscience
H3(13–32)R17me2aCi26(AB)	Ac-GKAPR[me2a]KQLATKAAR[Ci]KSAPAT-NH-Ahx-lysine-Biotin	2587.93	2588.93	518.59	AltaBioscience
H3(13–32)Ci17R26me2s(AB)	Ac-GKAPR[Ci]KQLATKAAR[me2s]KSAPAT-NH-Ahx-lysine-Biotin	2587.93	2588.93	518.59	AltaBioscience
H3(13–32)Ci17R26me2a(AB)	Ac-GKAPR[Ci]KQLATKAAR[me2a]KSAPAT-NH-Ahx-lysine-Biotin	2587.93	2588.93	518.59	AltaBioscience
H3(13–32)Ci17Ci26(AB)	Ac-GKAPR[Ci]KQLATKAAR[Ci]KSAPAT-NH-Ahx-lysine-Biotin	2560.88	2561.88	513.18	AltaBioscience
H4(1–20)(AB)	SGRGKGGKGLGKGGAKRHRK-NH-Ahx-lysine-Biotin	2457.86	2458.86	492.57	AltaBioscience
H4(1–20)R3me1(AB)	SGR[me1]GKGGKGLGKGGAKRHRK-NH-Ahx-lysine-Biotin	2471.88	2472.88	495.38	AltaBioscience
H4(1–20)R3me2s(AB)	SGR[me2s]GKGGKGLGKGGAKRHRK-NH-Ahx-lysine-Biotin	2485.89	2486.89	498.18	AltaBioscience
H4(1–20)R3me2a(AB)	SGR[me2a]GKGGKGLGKGGAKRHRK-NH-Ahx-lysine-Biotin	2485.89	2486.89	498.18	AltaBioscience
H4(1–20)Ci3(AB)	SGR[Ci]GKGGKGLGKGGAKRHRK-NH-Ahx-lysine-Biotin	2458.85	2459.85	492.77	AltaBioscience
H4(80–99)(AB)	Ac-TVTAMDVVYALKRQGRTLYG-NH-Ahx-lysine-Biotin	2749.89	2750.89	550.98	AltaBioscience
H4(80–99)R92me1(AB)	Ac-TVTAMDVVYALKR[me1]QGRTLYG-NH-Ahx-lysine-Biotin	2763.91	2764.91	553.78	AltaBioscience
H4(80–99)R92me2s(AB)	Ac-TVTAMDVVYALKR[me2s]QGRTLYG-NH-Ahx-lysine-Biotin	2777.92	2778.92	556.58	AltaBioscience
H4(80–99)R92me2a(AB)	Ac-TVTAMDVVYALKR[me2a]QGRTLYG-NH-Ahx-lysine-Biotin	2777.92	2778.92	556.58	AltaBioscience
H4(80–99)Ci92(AB)	Ac-TVTAMDVVYALKR[Ci]QGRTLYG-NH-Ahx-lysine-Biotin	2750.88	2751.88	551.18	AltaBioscience
H3(1–21)R2me2a	AR[me2a]TKQTARKSTGGKAPRKQLA	2280.36	2281.36	457.07	In house
H3(1–15)R9me2a	ARTKQTARR[me2a]STGGKA	1614.94	1615.94	323.99	In house
H3(1–21)R9me2a	ARTKQTARR[me2a]STGGKAPRKQLA	2308.37	2309.37	462.67	GL Biochem
H3(1–21)K4me3R9me2a	ARTK[me3]QTARR[me2a]STGGKAPRKQLA	2350.41	2351.41	471.08	GL Biochem
H3(1–15)K9me3	ARTKQTARK[me3]STGGKA	1600.95	1601.95	321.19	GL Biochem
H3(1–21)K4me3K9me3	ARTK[me3]QTARK[me3]STGGKAPRKQLA	2336.424	2337.42*	468.28	GL Biochem

Table S2 Peptides used. Peptides were synthesised as C-terminal amides. Peptides labelled (AB) with -NH-Ahx-K-biotin were synthesised with an aminohexanoic group, which was linked to N^{ε}-(D-biotin)-L-lysine-amide. Peptides produced in house were synthesised either using a Multipep RSi synthesiser (for screening) or a Liberty Blue automated microwave synthesizer. Those synthesised using the latter were purified to > 95% using a C18 column connected to HPLC and quantified using NMR. Purities and masses were then confirmed by LC–MS. Nomenclature: the initial letters represent the protein the peptide sequence is taken from; the numbers correspond to amino acid sequence numbers. K/R[X] corresponds to the modified lysine or arginine. Modifications are denoted as me1, me2, me3, for mono-, di-, and tri-methylations, respectively; me2a is asymmetric di-methyl arginine; me2s is symmetric di-methyl arginine; Ci is citrulline; and Ac represents acetylation. *H3(1–21)K4me3K9me3 was observed by MALDI-TOF MS as its $[MH]^{2+}$ ion, i.e. 1169.21 Da.

Enzyme	Cognate substrate	[Enzyme] (μM)	[Peptide] (μM)	[2OG] (μM)	[Ascorbate] (μM)	[$(NH_4)_2Fe(SO_4)_2$] (μM)	[TCEP] (mM)	Buffer pH7.5	
								[HEPES] (mM)	[NaCl] (mM)
KDM1A*	H3(1–21)K4me2 (12)	2	10	/	/	/	4	/	/
KDM3A	H3(1–21)K9me2 (13)	2	10	100	100	50	5	50	/
KDM3B	H3(1–21)K9me2 (13)	2	10	100	100	50	5	50	/
KDM4A	H3(1–21)K9me3 (14)	2	10	100	100	10	/	50	/
KDM4B	H3(1–21)K9me3 (14)	2	10	100	100	10	/	50	/
KDM4C	H3(1–21)K9me3 (14)	2	10	100	100	10	/	50	/
KDM4D	H3(1–21)K9me3 (14)	2	10	100	100	10	/	50	/
KDM4E	H3(1–21)K9me3 (15)	2	10	100	100	10	/	50	/
KDM5C	H3(1–21)K4me3 (16, 17)	2	10	100	100	10	/	50	/
KDM5D*	H3(1–21)K4me3 (17, 18)	2	10	100	100	10	5	50	50
KDM6B	H3(14–34)K27me3 (19)	2	10	100	100	10	5	50	50
KDM7A*	H3(1–21)K9me2 (20)	4	10	100	200	50	5	50	50

Table S3 Summary of MALDI–TOF MS assay conditions for enzymes optimised for substrate screening. *Assays were carried out at RT except for KDM1A, KDM5D, and KDM7A, at 37 °C. All reactions were quenched using 1% (v/v) HCOOH. Buffer used for Set7 and KDM1A assay: 20 mM Tris-HCl (pH 8.0), 0.01% (v/v) Triton X-100. These conditions enabled efficient turnover of established substrates.

A (%)	B (%)	Time (min)
100	0	0
100	0	1
50	50	1.2
5	95	3
5	95	4
100	0	4.10
100	0	6

Table S4 Conditions for LC–MS-based activity assays using Chromolith FastGradient RP-18 50-2 mm column (Merc) and a Xevo G2-XS Quadrupole Time-of-Flight (qTOF) MS machine. Total run time: 6 min. Solvent A: 0.1% (v/v) HCOOH in ddH_2O; Solvent B: 0.1% (v/v) HCOOH in acetonitrile.

A (%)	B (%)	Time (min)
95	5	0
95	5	0.5
76	24	1.5
70	30	2.5
68	32	6
15	85	7
15	85	7.5
95	5	8
95	5	10

Table S5 Conditions for calf thymus histone LC–MS-based activity assays using Waters BEH C4 reversed phase column (2.1 × 150 mm, 1.7 µm particle size, 300 Å pore size) with A Xevo G2-XS qTOF MS machine. Total run time: 10 min. Solvent A: 0.1% (v/v) HCOOH in ddH_2O; Solvent B 0.1% (v/v) HCOOH in acetonitrile.

Histone	A.A	PTM	KDM3A 515–1317	KDM4A 1–1,064	KDM4E 1–337	KDM5C 1–765	KDM6B 1141–1641
H3 (1–15)	R2	me1	–	/	+	+	–
		me2s	–	/	+	+	–
		me2a	–	+	+	+	–
	R8	me1	–	/	+	–	–
		me2s	–	/	+	+	–
		me2a	–	/	+	+	–
	<u>R4</u>	<u>me1</u>	/	/	/	+	/
		<u>me2s</u>	/	/	/	+	/
		<u>me2a</u>	/	/	/	+	/
	<u>R9</u>	<u>me1</u>	+	/	+	/	/
		<u>me2s</u>	+	/	+	/	/
		<u>me2a</u>	+	/	+	/	/
H3 (18–32)	R17	me1	–	/	–	–	–
		me2s	–	/	–	–	–
		me2a	–	/	–	–	–
	R26	me1	–	/	+	+	–
		me2s	–	/	+	+	–
		me2a	–	/	+	+	–
H3 (14–34)	<u>R27</u>	<u>me1</u>	/	/	/	/	/
		<u>me2s</u>	/	/	/	/	/
		<u>me2a</u>	/	/	/	/	+
H4 (1–15)	R3	me1	–	/	–	–	–
		me2s	–	/	+	+	–
		me2a	–	/	+	+	–

Table S6 Summary of results for incubations of histone H3 and H4 fragments with *N*-methylated arginines previously reported with KDM3A$^{515–1317}$, KDM4A$^{1–1,064}$, KDM4E$^{1–337}$, KDM5C$^{1–765}$, and KDM6B$^{1141–1,641}$. + = RDM activity observed, /= not tested, – = no activity observed (21). ‘a’ denotes asymmetric *N*-dimethylated arginine and ‘s’ denotes symmetric *N*-dimethylated arginine. Underlined residues and their methylation state denotes variants of the natural histone substrate of the KDMs.

Enzyme	H3 (1–15) R2me2a	H4 (1–15) R3me2a	G3BP1 (430–443) R435me2a	G3BP1 (431–451) R447me2a	G3BP1 (444–465) R447me2a	G3BP1 (444–465) R460me2a	FUS (488-513) R498me2a R503me2a	FUS (488-513) R498me2a	FUS (488-513) R503me2a
KDM1A	—	—	—	—	—	—	—	—	—
KDM3A	—	—	—	—	—	—	—	—	—
KDM3B	—	—	—	—	—	—	—	—	—
KDM4A	—*	—	—	—	—	—	—	—	—
KDM4B	—	—	—	—	—	—	—	—	—
KDM4D	+	—	—	—	—	—	—	—	—
KDM4E	++	+	—	—	—	—	—	—	—
KDM5C	++	++	—	+	++	++	—	—	—
KDM5D	++	—	—	—	+	+	—	—	—
KDM6B	—	—	—	—	—	—	—	—	—
KDM7A	—	—	—	—	—	—	—	—	—

Table S7 Summary of screen of histone and non-histone peptides with a panel of KDMs for RDM activity. Shifts in mass corresponding to removal of methyl groups (−14 Da) were observed with KDM4D (potentially), KDM4E, KDM5C, KDM5D. No mass shifts for KDM4A were observed with any of these peptides. — = no activity observed (< 10% demethylation/hydroxylation), ++ = new activity above ~10% demethylation (i.e., not previously reported in literature), + = potential new RDM activity with ~10% demethylation, ++ = previously reported activity observed above ~10% demethylation, + = previously reported activity with ~10% demethylation. n = 2 (independent assays). See Table S3 for assay conditions.

Histone	A.A	modification	KDM4A	KDM4D	KDM4E	KDM5C
H3 (1–21)	K4	me3	/	/	/	++
H3 (1–21)	K9	me3	++	++	++	/
H3 (1–20)	R2	/	—	—	—	—
		me1	—	—	++	++
		me2s	—	—	++	++
		me2a	—*	+	++	++
		Ci	—	—	—	—
	R8	me1	—	—	—	++
		me2s	—	—	++	++
		me2a	—	—	++	++
		Ci	—	—	—	—
H3 (13–32)	R17	me1	—	—	—	—
		me2s	—	—	—	—
		me2a	—	—	—	—
		Ci	—	—	—	—
	R26	me1	—	—	++	—
		me2s	—	—	++	—
		me2a	—	—	++	—
		Ci	—	—	—	—
H4 (1–20)	R3	/	—	—	—	—
		me1	—	+	—	++
		me2s	—	—	++	++
		me2a	—	+	++	++
		Ci	—	—	—	—
H4 (80–99)	R92	me1	—	—	—	—
		me2s	—	—	—	—
		me2a	—	—	—	—
		Ci	—	—	—	—

Table S8 Summary of results with unmodified, methylated, and citrullinated histone H3 and H4 peptides from the histone peptide library (AltaBiosciences Set 5) screened with KDM4A, KDM4D, KDM4E, and KDM5C. Demethylation (−14 Da shifts) in mass corresponding to removal of methyl groups, observed with KDM4D (potentially), KDM4E, and KDM5C. No mass shifts for KDM4A were observed with any of these peptides (except with the histone H3K9me3 positive control). — = no activity observed (< 10% demethylation), ++ = new activity above ~10% demethylation (i.e., not previously reported in literature), + = potential new activity at ~10% demethylation, ++ = previously reported activity observed above ~10% demethylation. n = 2 (technical duplicates). Ci; citrulline. See Table S3 for assay conditions.

Protein	*N*-term A.A. #			Potential substrate			*C*-term A.A. #
H3	1		A	**R***	T	K	4
	6	T	A	**R***	K	S	10
	15	A	P	**R**	K	Q	19
	24	A	A	**R***	K	S	28
H4	1	S	G	**R***	G	K	5
	90	L	K	**R**	Q	G	94
G3BP1	433	G	P	**R**	G	G	437
	445	P	P	**R***	G	G	449
	458	V	G	**R***	G	L	462
FUS	496	G	G	**R**	G	G	500
	501	G	D	**R**	G	G	505

Table S9 Comparison of sequences surrounding potential arginine substrates of the KDMs. Amino acid residues are coded in colours corresponding to the properties of their side chains. **Green**: hydrophobic: aliphatic, **blue**: hydrophilic: polar uncharged, **purple**: hydrophilic: acidic, and **red**: hydrophilic: basic. *denotes substrates of the JmjC-KDMs (at least with the assay conditions used).

References

1. Shi, Y., Lan, F., Matson, C., Mulligan, P., Whetstine, J. R., Cole, P. A., Casero, R. A., and Shi, Y. (2004) Histone Demethylation Mediated by the Nuclear Amine Oxidase Homolog LSD1. *Cell*. **119**, 941–953
2. Rose, N. R., Woon, E. C., Tumber, A., Walport, L. J., Chowdhury, R., Li, X. S., King, O. N., Lejeune, C., Ng, S. S., Krojer, T., Chan, M. C., Rydzik, A. M., Hopkinson, R. J., Che, K. H., Daniel, M., Strain-Damerell, C., Gileadi, C., Kochan, G., Leung, I. K., Dunford, J., Yeoh, K. K., Ratcliffe, P. J., Burgess-Brown, N., von Delft, F., Muller, S., Marsden, B., Brennan, P. E., McDonough, M. A., Oppermann, U., Klose, R. J., Schofield, C. J., and Kawamura, A. (2012) Plant growth regulator daminozide is a selective inhibitor of human KDM2/7 histone demethylases. *J Med Chem*. **55**, 6639–6643
3. Aszykowska, S. (2013) 2L Purification of JMJD1AA-c223 [JMJD1AA-p043]. [online] https://www.thesgc.org/sites/default/files/construct-data/2l-purification-JMJD1AA-c223.pdf (Accessed September 26, 2020)
4. Ng, S. S., Kavanagh, K. L., McDonough, M. A., Butler, D., Pilka, E. S., Lienard, B. M. R., Bray, J. E., Savitsky, P., Gileadi, O., Von Delft, F., Rose, N. R., Offer, J., Scheinost, J. C., Borowski, T., Sundstrom, M., Schofield, C. J., and Oppermann, U. (2007) Crystal structures of histone demethylase JMJD2A reveal basis for substrate specificity. *Nature*. **448**, 87–91
5. Rose, N. R., Ng, S. S., Mecinović, J., Liénard, B. M. R., Bello, S. H., Sun, Z., McDonough, M. A., Oppermann, U., and Schofield, C. J. (2008) Inhibitor Scaffolds for 2-Oxoglutarate-Dependent Histone Lysine Demethylases. *J. Med. Chem.* **51**, 7053–7056
6. Tumber, A., Nuzzi, A., Hookway, E. S., Hatch, S. B., Velupillai, S., Johansson, C., Kawamura, A., Savitsky, P., Yapp, C., Szykowska, A., Wu, N., Bountra, C., Strain-Damerell, C., Burgess-Brown, N. A., Ruda, G. F., Fedorov, O., Munro, S., England, K. S., Nowak, R. P., Schofield, C. J., La Thangue, N. B., Pawlyn, C., Davies, F., Morgan, G., Athanasou, N., Muller, S., Oppermann, U., and Brennan, P. E. (2017) Potent and Selective KDM5 Inhibitor Stops Cellular Demethylation of H3K4me3 at Transcription Start Sites and Proliferation of MM1S Myeloma Cells. *Cell Chem Biol*. **24**, 371–380
7. Johansson, C., Velupillai, S., Tumber, A., Szykowska, A., Hookway, E. S., Nowak, R.

P., Strain-Damerell, C., Gileadi, C., Philpott, M., Burgess-Brown, N., Wu, N., Kopec, J., Nuzzi, A., Steuber, H., Egner, U., Badock, V., Munro, S., LaThangue, N. B., Westaway, S., Brown, J., Athanasou, N., Prinjha, R., Brennan, P. E., and Oppermann, U. (2016) Structural analysis of human KDM5B guides histone demethylase inhibitor development. *Nat Chem Biol*. **12**, 539–545

8. Belle, R. (2019) *Histone Lysine and DNA Methylation: Dynamic Marks in the Chromatin*. Ph.D. thesis, Oxford
9. Coleman, O. D. (2018) *Development, Synthesis and Characterisation of Chemical Probes for PHD Fingers*. Ph.D. thesis, Oxford
10. Zhang, Y. (2019) *Investigating the substrate selectivity and the inhibitor of the human KDM7 sub-family of JmjC histone lysine demethylases*. Ph.D. thesis, Oxford
11. Lizcano, J. M., Unzeta, M., and Tipton, K. F. (2000) A spectrophotometric method for determining the oxidative deamination of methylamine by the amine oxidases. *Anal. Biochem.* **286**, 75–79
12. Forneris, F., Binda, C., Vanoni, M. A., Battaglioli, E., and Mattevi, A. (2005) Human histone demethylase LSD1 reads the histone code. *J Biol Chem*. **280**, 41360–41365
13. Yamane, K., Toumazou, C., Tsukada, Y. ichi, Erdjument-Bromage, H., Tempst, P., Wong, J., and Zhang, Y. (2006) JHDM2A, a JmjC-Containing H3K9 Demethylase, Facilitates Transcription Activation by Androgen Receptor. *Cell*. **125**, 483–495
14. Whetstine, J. R., Nottke, A., Lan, F., Huarte, M., Smolikov, S., Chen, Z., Spooner, E., Li, E., Zhang, G., Colaiacovo, M., and Shi, Y. (2006) Reversal of Histone Lysine Trimethylation by the JMJD2 Family of Histone Demethylases. *Cell*. **125**, 467–481
15. Hillringhaus, L., Yue, W. W., Rose, N. R., Ng, S. S., Gileadi, C., Loenarz, C., Bello, S. H., Bray, J. E., Schofield, C. J., and Oppermann, U. (2011) Structural and evolutionary basis for the dual substrate selectivity of human KDM4 histone demethylase family. *J Biol Chem*. **286**, 41616–41625
16. Christensen, J., Agger, K., Cloos, P. A. C., Pasini, D., Rose, S., Sennels, L., Rappsilber, J., Hansen, K. H., Salcini, A. E., and Helin, K. (2007) RBP2 Belongs to a Family of Demethylases, Specific for Tri-and Dimethylated Lysine 4 on Histone 3. *Cell*. **128**, 1063–1076
17. Iwase, S., Lan, F., Bayliss, P., de la Torre-Ubieta, L., Huarte, M., Qi, H. H., Whetstine, J. R. R., Bonni, A., Roberts, T. M., and Shi, Y. (2007) The X-Linked Mental Retardation Gene SMCX/JARID1C Defines a Family of Histone H3 Lysine 4 Demethylases. *Cell*. **128**, 1077–1088

18. Lee, M. G., Norman, J., Shilatifard, A., and Shiekhattar, R. (2007) Physical and Functional Association of a Trimethyl H3K4 Demethylase and Ring6a/MBLR, a Polycomb-like Protein. *Cell.* **128**, 877–887

19. Hong, S. H., Cho, Y. W., Yu, L. R., Yu, H., Veenstra, T. D., and Ge, K. (2007) Identification of JmjC domain-containing UTX and JMJD3 as histone H3 lysine 27 demethylases. *Proc. Natl. Acad. Sci. U. S. A.* **104**, 18439–18444

20. Tsukada, Y. I., Ishitani, T., and Nakayama, K. I. (2010) KDM7 is a dual demethylase for histone H3 Lys 9 and Lys 27 and functions in brain development. *Genes Dev.* **24**, 432–437

21. Walport, L. J., Hopkinson, R. J., Chowdhury, R., Schiller, R., Ge, W., Kawamura, A., and Schofield, C. J. (2016) Arginine demethylation is catalysed by a subset of JmjC histone lysine demethylases. *Nat. Commun.* **7**, 11974
